# On the origin of the stereoselectivity in chiral amide-based ammonium ylide-mediated epoxidations

**DOI:** 10.1007/s00706-016-1866-8

**Published:** 2016-11-24

**Authors:** Johanna Novacek, Raphaël Robiette, Mario Waser

**Affiliations:** 1 Institute of Organic Chemistry, Johannes Kepler University Linz, Altenbergerstraße 69, 4040 Linz, Austria; 2 Institute of Condensed Matter and Nanosciences, Université catholique de Louvain, Place Louis Pasteur 1 box L4.01.02, 1348 Louvain-la-Neuve, Belgium

**Keywords:** Mechanistic studies, DFT calculations, Absolute configuration, Auxiliaries

## Abstract

**Abstract:**

Detailed DFT studies provide an in-depth mechanistic understanding for the use of chiral amide-based ammonium ylides in epoxidation reactions. It is shown that the used chiral auxiliary efficiently shields one face of the ylide, which thus results in an extraordinarily high stereoselectivity giving only one trans-isomer with perfect control of the absolute configuration.

**Graphical abstract:**

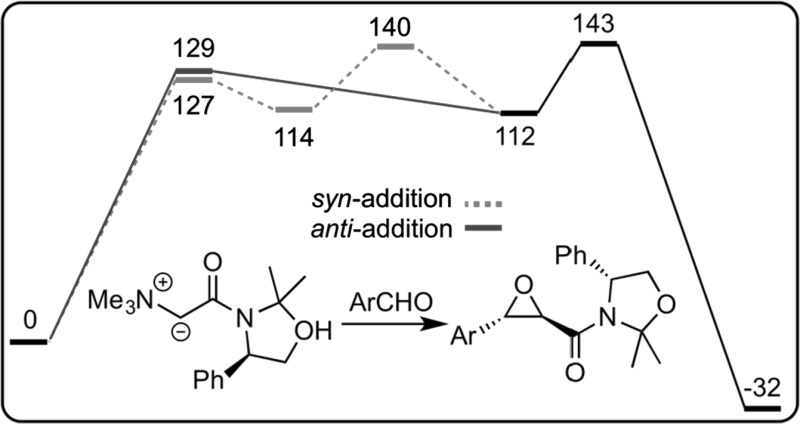

**Electronic supplementary material:**

The online version of this article (doi:10.1007/s00706-016-1866-8) contains supplementary material, which is available to authorized users.

## Introduction

The use of easily available ammonium ylides [1] has emerged as a powerful alternative to classically used sulfonium ylides [2] for the synthesis of three-membered ring compounds like epoxides [3–11], aziridines [12, 13], and cyclopropanes [14–16]. These reactions usually benefit from very high and predictable diastereoselectivities and broad application scopes [3–16], as illustrated for epoxidation reactions using amide-based ylides **1** with different aldehydes **2** (Scheme 1a) [7]. In addition, the use of chiral amines often allows for highly enantioselective protocols. However, some limitations were observed: While the use of Cinchona alkaloids as chiral amine leaving groups was found to be a very versatile strategy for enantioselective cyclopropanation reactions [14, 15], these simple naturally occurring chiral amines were not suited for epoxidations and aziridinations [6, 11, 13]. Our group has for years been interested in the development of new catalytic [17, 18] and auxiliary-based methods [8, 11] for the synthesis of chiral heterocycles. Based on this general interest, we recently carried out an extensive screening and optimization of different chiral amines for ammonium ylide-mediated epoxidation reactions. This allowed us to overcome the above mentioned obstacles when using Cinchona alkaloids and to develop the first highly enantioselective and high yielding ammonium ylide **4** mediated epoxidation protocol (Scheme 1b) [11]. Alternatively, very high stereoselectivities were also obtained by using chiral amide-based ylides **5**, which resulted in complete control of the absolute and relative configuration (Scheme 1c) [8].

**Figure:**
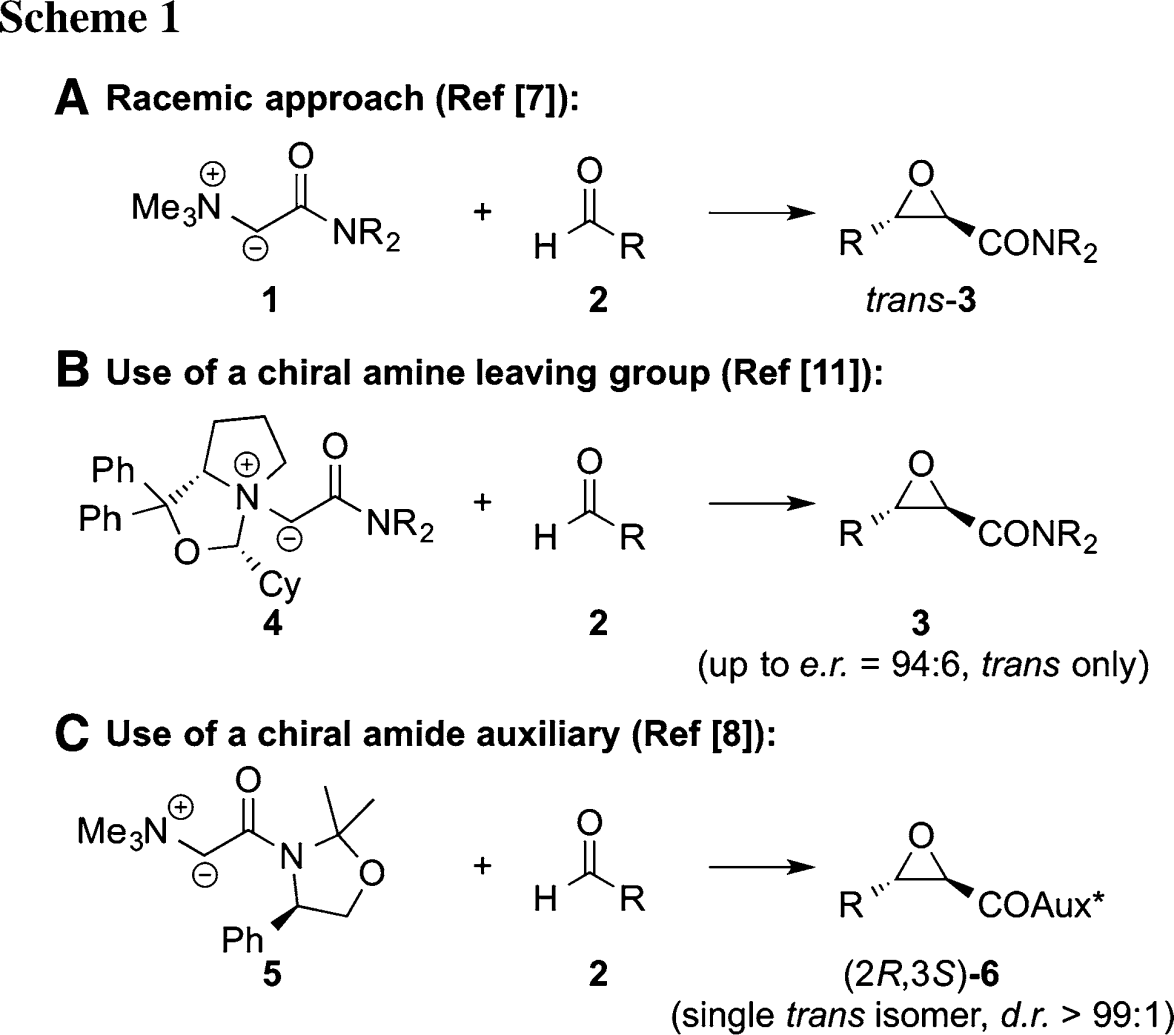

Impressed by the extraordinarily high stereoselectivities obtained using the chiral phenylglycinol-derived auxiliary-containing ylides **5** [8] and based on our recently gathered mechanistic understanding of the analogous racemic reactions with ylides **1** [11], we have now performed detailed DFT calculations for the reaction of **5** with benzaldehyde (**2**) to elucidate the origin of this high stereoselectivity.

## Results and discussion

We have recently shown for racemic approaches that the reaction proceeds via a *syn*-addition of ylide **1** to aldehyde **2** in a [2+2] approach to give a *cisoid* betaine intermediate (**A**). This preference for a *cisoid* geometry of the transition state (TS) is accounted for by the stabilizing Coulombic interactions between the negatively charged oxygen and the positively charged nitrogen atom in such a geometry (for similar observations with oniom ylides see Ref [19]). This step is followed by torsional rotation to yield the *transoid* conformer **B**, and finally ring closure (elimination of the amine) to give the corresponding epoxide **3** (Scheme 2). The observed high *trans*-diastereoselectivity was found to be a consequence of the more favoured elimination from *trans* betaine (**B**) as compared to the analogous *cis*-pathway [11].

**Figure:**
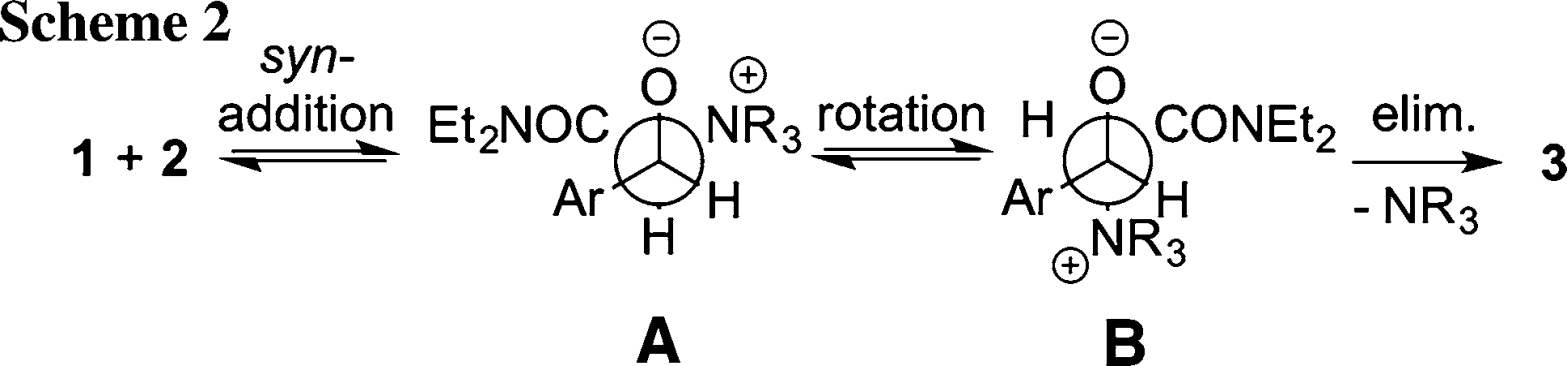

We now investigated how the additional steric demand of the auxiliary of **5** influences this pathway using DFT methods in order to understand the origin of the extraordinarily high diastereoselectivity of the reaction of **5** with **2**. According to the experimental selectivity, i.e. exclusive formation of the (2*R*,3*S*)-configured epoxides **6**, attack of the *Si*-face of the ylide to the *Re*-face of the aldehyde must be the preferred pathway. We have investigated the free energy profiles for all four possible scenarios of the reaction between ylide **5** and benzaldehyde **2**: a) *Si*_ylide_ → *Re*_Ald_ giving *trans*-(2*R,*3*S*)-**6**; b) *Si*_ylide_ → *Si*_Ald_ giving *cis*-(2*R*,3*R*)-**6**; c) *Re*_ylide_ → *Si*_Ald_ giving *trans*-(2*S*,3*R*)-**6**; d) *Re*_ylide_ → *Re*_Ald_ giving *cis*-(2*S*,3*S*)-**6**. Calculations were carried out at the B3LYP-D3/6-311+G**//B3LYP-D3/6-31G* level of theory [20, 21] including a continuum description of dichloromethane as solvent.

**Fig. 1:**
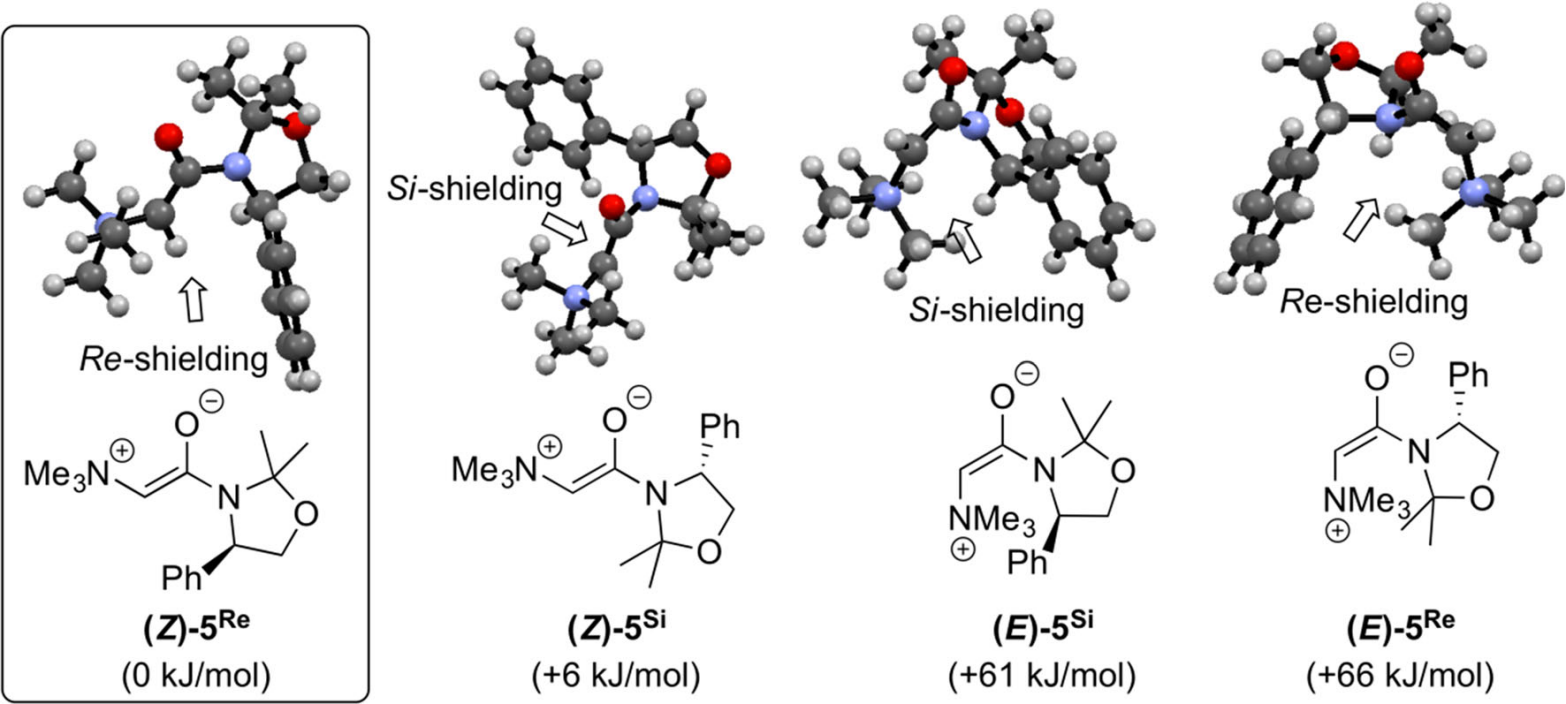
Calculated structures of the four most relevant ylide conformers **5**

First, the ylide conformations were explored (the optimized structures have to some extent been reported in Ref. [8] before). Four conformers are possible, two with a *Z*-configuration and two with an *E*-configuration (Fig. 1). The *E-*configured ylides were found to lye higher in free energy (at least 60 kJ/mol) than those with a *Z*-configuration. This can be accounted for by the stabilizing Coulombic interactions between the ammonium group and the amide-oxygen in the case of a *Z*-configuration, which in contrast is absent in the *E*-isomers. Among the *Z*-configured ylides, the most stable is the one with the *Re*-face being shielded ((*Z*)-**5** ^**Re**^); the other one with the shielded *Si*-face ((*Z*)-**5** ^**Si**^) lying 6 kJ/mol higher in free energy. To make sure that only the more stable ylide conformer (*Z*)-**5** ^**Re**^ is relevant for the investigated pathways, the free energy of the transition states were calculated for the addition of both *Z*-ylides to aldehydes. It was found that the addition from ylide (*Z*)-**5** ^**Re**^ was thoroughly more favoured.

**Fig. 2:**
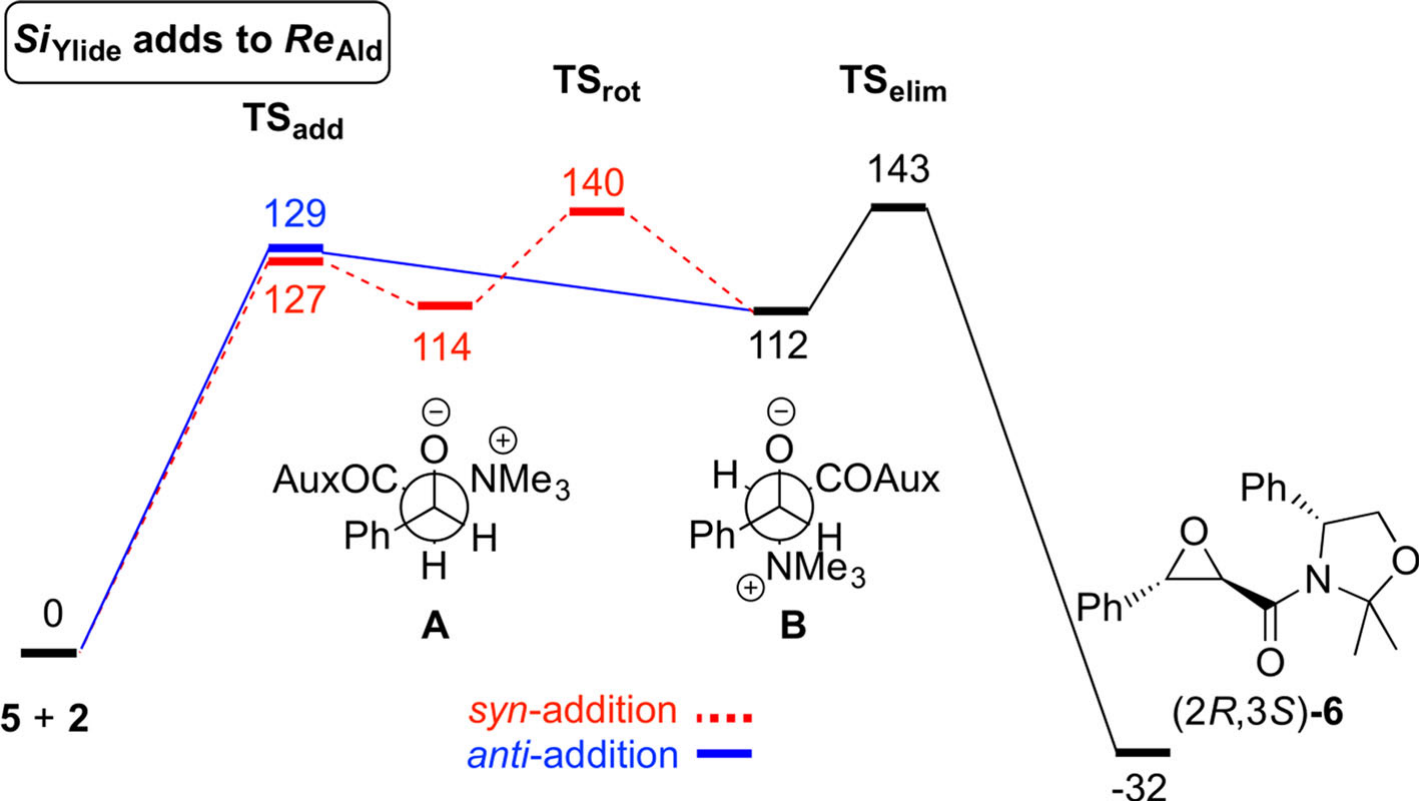
Calculated free energy profile for the major pathway leading to *trans*-(2*R*,3*S*)-epoxide **6** (relative free energies are given in kJ/mol)

The addition of the *Si*-face of the ylide to the *Re*-face of the aldehyde towards formation of the major *trans*-(2*R*,3*S*)-epoxide **6** was first computed (Fig. 2). In striking contrast to the achiral scenario with ylides **1**, which proceed via a *syn*-addition followed by bond rotation, the *syn*- and *anti*-additions of ylide **5** are almost isoenergetic. However, the rotational transition state (from *cisoid* betaine **A** to *transoid* betaine **B**) is destabilized because of an eclipsed conformation of the ammonium group and the phenyl group (coordinates are given in the supporting information). It results that *syn*-addition is not the most favored pathway herein. As for the achiral ammonium ylides **1**, the elimination transition state is the highest point on the free energy profile, and hence the rate-determining step.

By computing the other three addition possibilities we found a very pronounced difference between pathways leading to *cis*- and those leading to *trans*-epoxides (Table 1). These results show that the pathway involving an *anti*-addition (direct formation of **B**) is preferred for both *trans*-epoxides whereas for *cis*-epoxide formation it is the pathway involving a *syn*-addition to give **A** first and then rotation to **B**, which is favored.

**Table 1:** Computed relative free energies (kJ/mol) for the formation of all four possible diastereomeric epoxides **6** (B3LYP-D3/6-311+G**(dichloromethane)//B3LYP-D3/6-31G*(dichloromethane))

Pathway	TS_add_	A	TS_rot_	B	TS_elim_	6
*Si*_ylide_ → *Re*_Ald_ giving *trans*-(2*R,*3*S*)-**6**						
Syn-add.	127	114	140	112	143	−32
Anti-add.	129	–	–			
*Si*_ylide_ → *Si*_Ald_ giving *cis*-(2*R,*3*R*)-**6**						
Syn-add.	123	103	122	120	157	−22
Anti-add.	137	–	–			
*Re*_ylide_ → *Si*_Ald_ giving *trans*-(2*S*,3*R*)-**6**						
Syn-add.	140	129	169	140	152	−38
Anti-add.	159	–	–			
*Re*_ylide_ → *Re*_Ald_ giving *cis*-(2*S*,3*S*)-**6**						
Syn-add.	143	118	178	158	178	−16
Anti-add.	179	–	–			

Relative free energies are referred to the starting materials **2** and **5**

**Fig. 3:**
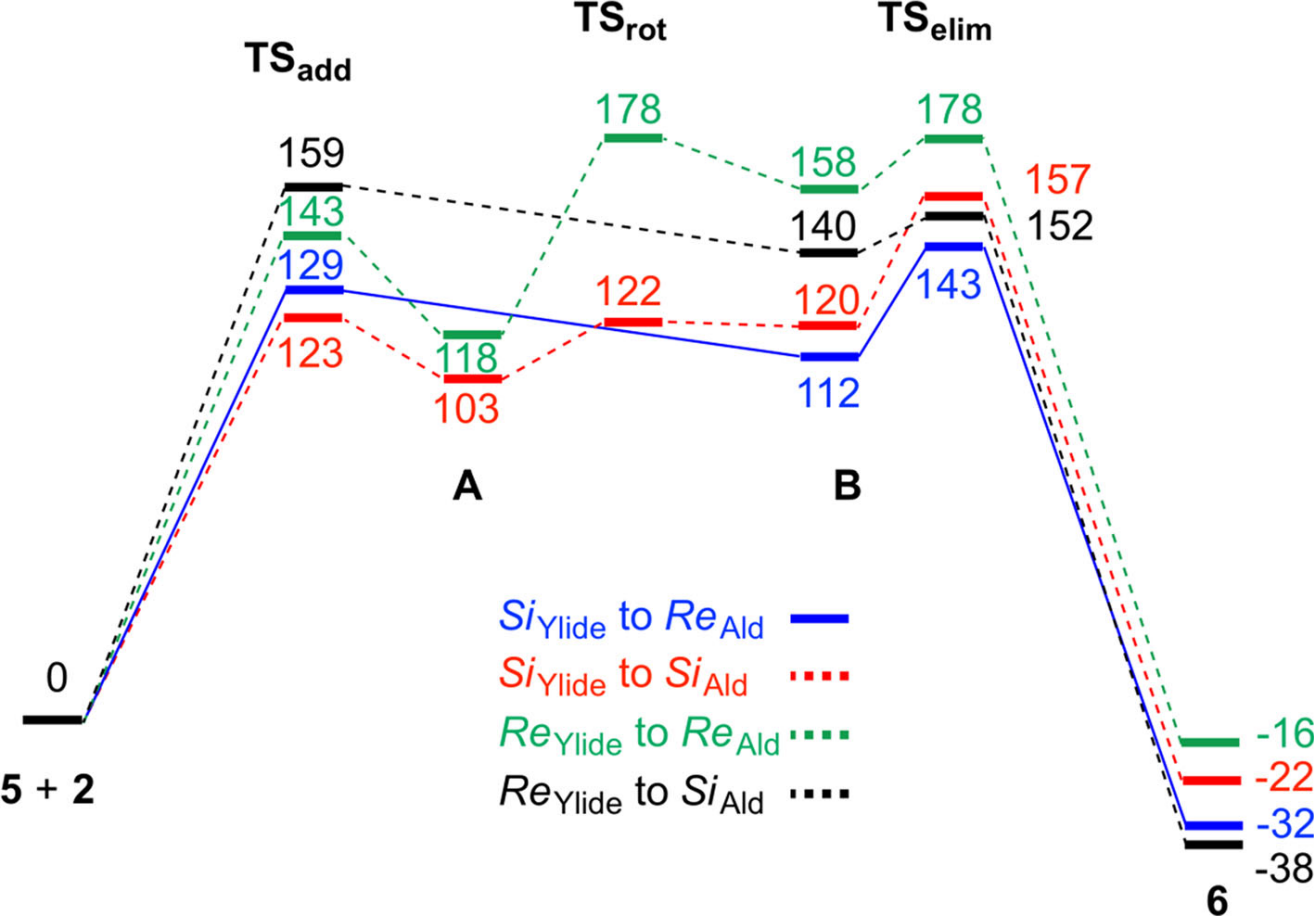
Pathways leading to the four possible stereoisomers of epoxide **6** (relative free energies are given in kJ/mol)

The exclusive formation of *trans*-(2*R*,3*S*)-epoxide **6** (via the *Si*_Ylide_ *Re*_Ald_ pathway) can be understood by comparing the lowest free energy pathways towards each stereoisomer (Fig. 3). Indeed, these free energy profiles show that TS_add_ and *transoid* betaines (**B**) formation are highly disfavored in the case of a *Re*_Ylide_ approach. This is partially compensated by a faster elimination step, as compared to *Si*_Ylide_ cases, but not totally. Concerning the two *Si*_Ylide_ pathways, the free energy barrier to elimination is rather similar in both cases (31 and 37 kJ/mol for *Si*_Ylide_ *Re*_Ald_ and *Si*_Ylide_ *Si*_Ald_, pathways respectively) but *transoid* betaine (**B**) formation being more favored (by 14 kJ/mol) for the *trans* isomer (*Si*_Ylide_ *Re*_aldehyde_ pathway) that is this latter which is preferentially formed; explaining thus the exclusive formation of *trans*-(2*R*,3*S*)-epoxide **6**.

## Conclusion

The DFT studies carried out herein provide a clear rationale for the very high stereoselectivities obtained when using chiral amide-based ammonium ylides for epoxidation reactions. We show that the shielding of the *Re*-face of the used ylide plays a crucial role by controlling both kinetically and thermodynamically the *transoid* betaine formation, favoring betaines involving a *Si*_Ylide_ approach. A second important factor is the higher stability of the *trans* stereoisomer of *transoid* betaines (**B**) as compared to its *cis* isomer. It is worth noting also that in contrast to the recently investigated use of achiral ylides, these chiral amide auxiliaries lead to a direct *anti* addition of the ylide to the aldehyde for *trans*-epoxides formation.

## Methods

Geometry optimization has been performed using the Jaguar 8.0 pseudospectral program package using the well-established B3LYP hybrid density functional and the standard split valence polarized 6-31G* basis as implemented in Jaguar. All the optimization calculations include an implicit description of dichloromethane solvent using the Poisson–Boltzmann polarizable continuum method as incorporated in Jaguar, and parameters for dichloromethane. Electronic energies were obtained by single point calculations at the B3LYP-D3/6-311 + G**(dichloromethane) level of theory. The correct nature of each stationary point (minima or transition state) has been checked by performing frequency calculations at the B3LYP-D3/6-31G*(dichloromethane) level of theory. Thermal and entropic contributions to free energy (at 298.15 K) and zero-point energy have been obtained from these frequency calculations. The free energy values were corrected by a concentration term, equal to RT ln (V_mol_gas_1 atm/V_mol_1 M), i.e. 7.9 kJ/mol at 298.15 K.

For the large reaction systems there are usually several local minima or saddle points corresponding to each intermediate or transition state. We have made a systematic attempt to locate all possible local minima and saddle points, with the data presented referring to the lowest energy form unless mentioned otherwise. All species have been fully geometry optimized, and the Cartesian coordinates are supplied in the supporting information.

## Electronic supplementary material

Below is the link to the electronic supplementary material.

Supplementary material 1 (PDF 257 kb)
