# Supplementary material for: On the origin of the stereoselectivity in chiral amide-based ammonium ylide-mediated epoxidations
Source: Monatsh Chem. 2016 Nov 24;148(1):77–81. doi: 10.1007/s00706-016-1866-8 (PMC5225234; doi:10.1007/s00706-016-1866-8)
Supplement: Supplementary file 1 — Supplementary material 1 (PDF 257 kb) [file 706_2016_1866_MOESM1_ESM.pdf]

On the origin of the stereoselectivity in chiral amide-based ammonium ylide-mediated epoxidations

Johanna Novacek ● Raphaël Robiette ● Mario Waser

Computational methods

Geometry optimization has been performed using the Jaguar 8.0 pseudospectral program package using the well-established B3LYP hybrid density functional and the standard split valence polarized 6-31G*-D3 basis as implemented in Jaguar.

All the optimization calculations include an implicit description of dichloromethane solvent using the Poisson–Boltzmann polarizable continuum method as incorporated in Jaguar, and parameters for dichloromethane.

Electronic energies were obtained by single point calculations at the B3LYP-D3/ 6-311+G**(dichloromethane) level of theory.

The correct nature of each stationary point (minima or transition state) has been checked by performing frequency calculations at the B3LYP/6-31G*-D3(dichloromethane) level of theory.

Thermal and entropic contributions to free energy (at 298.15 K) and zero-point energy have been obtained from these frequency calculations. In Jaguar, the translational partition function is computed for ideal gas standard conditions, corresponding to a pressure of 1 atmosphere at 298.15 K. For solution reactions, the standard condition is instead 1 mol/l. Accordingly, the free energy value computed in Jaguar was corrected by a concentration term, equal to RT ln (V_mol_gas_1atm / V_mol_1M), i.e. 1.89 kcal/mol at 298.15 K.

For the large reaction systems there are usually several local minima or saddle points corresponding to each intermediate or transition state. We have made a systematic attempt to locate all possible local minima and saddle points, with the data presented referring to the lowest energy form unless mentioned otherwise. All species have been fully geometry optimized, and the Cartesian coordinates are supplied.

It has to be mentioned that, in some cases, additional low imaginary frequencies (negative eigenvalues) were obtained. This, most probably, results from numerical errors due to the fact that calculations of vibrational frequencies in the presence of continuum solvent can only be done semi-numerically in Jaguar (numerical differentiation of analytical gradients). We thus have further confirmed the correct nature of all TSs by animating the normal mode of the (larger) imaginary frequency.

PhCHO

E(B3LYP-D3/6-31G*(dichloromethane)) = -345.58202839847
E(B3LYP-D3/6-311+G**(dichloromethane)) = -345.67900173549
G_{tot}(B3LYP-D3/6-31G*(dichloromethane)) = -345.501970

Number of imaginary frequencies: 0

C1	-1.08980	0.82310	0.00000	C6	-0.40100	2.03680	0.00000	H11	-0.94980	2.97560	0.00000
C2	-0.37920	-0.38800	0.00000	H7	-2.17930	0.80970	0.00000	C12	-1.11870	-1.66760	0.00000
C3	1.02760	-0.37780	0.00000	H8	1.56280	-1.32420	0.00000	O13	-0.59700	-2.77540	0.00000
C4	1.71250	0.83440	0.00000	H9	2.80030	0.84540	0.00000	H14	-2.22530	-1.56690	0.00000
C5	0.99850	2.04140	0.00000	H10	1.53580	2.98760	0.00000				

Z-Ylide (*Re*-shielding)

E(B3LYP-D3/6-31G*(dichloromethane)) = -884.05888389799
E(B3LYP-D3/6-311+G**(dichloromethane)) = -884.30432373979
G_{tot}(B3LYP-D3/6-31G*(dichloromethane)) = -883.721283

Number of imaginary frequencies: 0

C1	2.40500	7.82370	13.76970	O16	3.57130	4.71170	18.33590	H31	-0.64720	8.99600	20.25200
H2	1.77340	7.50810	12.94130	C17	4.06210	5.58800	17.33150	H32	-2.20870	9.31980	19.42250
O3	-0.41310	6.11550	18.13830	C18	1.53020	3.66770	17.51380	C33	-1.28850	8.72780	19.41110
N4	1.85360	6.11950	17.69750	H19	1.75470	2.69650	17.97140	C34	-1.41970	8.74130	16.95150
C5	3.29980	8.88520	13.60260	H20	1.93980	3.68650	16.49770	H35	-1.66190	7.67930	16.96870
C6	2.31900	7.16500	14.99630	H21	0.44830	3.80870	17.45730	H36	-2.32280	9.35490	17.02400
H7	1.62300	6.34290	15.13580	C22	1.71130	4.74210	19.80940	H37	-0.85880	8.98830	16.04960
C8	3.12170	7.55920	16.07390	H23	2.14400	5.58990	20.35100	C38	-0.14370	10.46690	18.13010
C9	3.10860	6.79370	17.38560	H24	2.08280	3.81160	20.25350	H39	-1.04620	11.07500	18.22420
H10	3.42560	7.46670	18.20150	H25	0.62470	4.78090	19.89370	H40	0.53230	10.65340	18.96790
C11	4.01020	8.62620	15.90260	C26	0.72950	8.20050	18.08830	H41	0.36110	10.69250	17.18830
H12	4.63660	8.94220	16.73610	C27	0.64510	6.81170	17.98600	H42	5.10020	5.84600	17.56210
C13	4.10280	9.28490	14.67340	N28	-0.52230	9.01280	18.13940	H43	4.02330	5.12210	16.33480
H14	4.80200	10.10950	14.55270	H29	1.57760	8.77940	17.76340	H44	3.36950	9.39660	12.64490
C15	2.13020	4.80620	18.34160	H30	-1.50090	7.66000	19.42300				

Z-Ylide (*Si*-shielding)

E(B3LYP-D3/6-31G*(dichloromethane)) = -884.05486148219
E(B3LYP-D3/6-311+G**(dichloromethane)) = -884.30114848302
G_{tot}(B3LYP-D3/6-31G*(dichloromethane)) = -883.718214

Number of imaginary frequencies: 0

C1	2.98380	7.91790	13.74860	C15	1.89970	4.81890	18.13810	H29	-0.55220	6.01610	16.34700
H2	2.48530	7.63940	12.82170	O16	3.31730	4.66310	18.37030	H30	-0.79450	9.27950	17.69120
O3	0.86280	7.93830	18.69050	C17	3.99440	5.52940	17.46640	H31	-1.39660	8.94430	16.03970
N4	1.77010	6.13770	17.45350	C18	1.41380	3.69770	17.21200	H32	-2.55630	9.41520	17.33130
C5	3.91180	8.96440	13.75670	H19	1.55440	2.71610	17.68090	C33	-1.62440	8.88640	17.10510
C6	2.69370	7.22760	14.92690	H20	1.96820	3.72440	16.26760	C34	-2.14650	7.31490	18.92700
H7	1.96830	6.41900	14.93450	H21	0.35010	3.83590	16.99020	H35	-1.30790	7.70040	19.50550
C8	3.32340	7.57180	16.13000	C22	1.22320	4.75410	19.50680	H36	-3.05540	7.89520	19.11330
C9	3.08840	6.76630	17.39460	H23	1.54970	5.59520	20.12380	H37	-2.31190	6.25960	19.15090
H10	3.26800	7.40430	18.27260	H24	1.48930	3.81400	20.00190	C38	-2.87130	6.82370	16.64150
C11	4.24420	8.62700	16.13160	H25	0.13640	4.79980	19.39390	H39	-3.80370	7.35500	16.84940
H12	4.73410	8.90800	17.06300	C26	-0.50310	6.68260	17.19290	H40	-2.61260	6.92050	15.58390
C13	4.54120	9.31770	14.95330	C27	0.67010	7.01360	17.84310	H41	-2.96840	5.76760	16.90340
H14	5.26250	10.13280	14.96970	N28	-1.76900	7.42370	17.46760	H42	4.99230	5.74010	17.86210

H43	4.09550	5.07030	16.47040	H44	4.14050	9.50160	12.83820

E-Ylide (*Re*-shielding)

E(B3LYP-D3/6-31G*(dichloromethane)) = -884.03642842508
E(B3LYP-D3/6-311+G**(dichloromethane)) = -884.28290707568
Gtot(B3LYP-D3/6-31G*(dichloromethane)) = -883.695308

Number of imaginary frequencies: 1 (-27.95)

C1	4.84930	6.66760	14.07570	O16	3.76670	5.15030	18.16160	H31	-1.38900	7.10640	18.40430
H2	5.56420	5.96810	13.64580	C17	3.88470	6.55890	18.35510	N32	-1.07080	6.45790	16.38030
O3	0.97090	7.10100	19.43920	C18	2.45850	3.85100	16.65850	C33	-0.44520	7.45260	15.42740
N4	1.76420	6.15250	17.40490	H19	3.20280	3.08050	16.88750	H34	0.62980	7.28100	15.43250
C5	4.42430	7.77580	13.33570	H20	2.74740	4.35600	15.73330	H35	-0.85300	7.30640	14.42220
C6	4.36730	6.45440	15.36880	H21	1.49680	3.35160	16.50470	H36	-0.67190	8.45620	15.79030
H7	4.71560	5.59780	15.93770	C22	1.71270	4.19600	19.03490	C37	-0.78160	5.06890	15.87280
C8	3.44510	7.34130	15.94790	H23	1.79000	4.84070	19.90990	H38	0.29670	4.95850	15.84010
C9	2.88780	7.14400	17.35050	H24	2.20510	3.23830	19.23720	H39	-1.22000	4.35200	16.56930
H10	2.50740	8.10120	17.71160	H25	0.65360	4.00820	18.82980	H40	-1.21170	4.95070	14.87380
C11	3.04290	8.45860	15.20200	C26	-0.61100	6.67510	17.78960	C41	-2.56580	6.65470	16.35150
H12	2.35100	9.17410	15.64420	C27	0.68450	6.67250	18.28360	H42	-2.92290	6.49430	15.33200
C13	3.52070	8.67490	13.90600	H28	3.58830	6.84300	19.37230	H43	-3.02300	5.93500	17.03270
H14	3.19360	9.54990	13.34630	H29	4.92740	6.83940	18.17370	H44	-2.79190	7.67350	16.67240
C15	2.38640	4.84790	17.82410	H30	4.80180	7.94080	12.32830				

E-Ylide (*Si*-shielding)

E(B3LYP-D3/6-31G*(dichloromethane)) = -884.03383512892
E(B3LYP-D3/6-311+G**(dichloromethane)) = -884.28220903416
G_{tot}(B3LYP-D3/6-31G*(dichloromethane)) = -883.695300

Number of imaginary frequencies: 0

C1	2.00780	8.24400	14.44600	O16	3.59370	4.15730	17.93010	H31	-0.38130	8.02100	19.06620
H2	1.15370	8.21150	13.77250	C17	4.17180	5.27040	17.26040	N32	1.47930	8.16810	20.11930
O3	-0.24060	5.90650	17.69680	C18	1.63970	3.86790	16.51440	C33	2.38500	9.24680	19.55860
N4	2.04940	5.81310	18.09590	H19	1.87190	2.80390	16.38630	H34	3.03250	8.81080	18.80400
C5	3.06410	9.12290	14.19200	H20	2.09690	4.42650	15.69180	H35	1.75060	10.00230	19.09250
C6	2.03620	7.40440	15.56260	H21	0.56240	4.02820	16.47010	H36	2.98200	9.69030	20.36240
H7	1.20410	6.73630	15.76350	C22	1.55370	3.56780	19.02590	C37	2.28990	7.14080	20.87290
C8	3.12610	7.43630	16.44130	H23	2.00450	3.88570	19.97100	H38	2.88210	6.56200	20.17120
C9	3.26080	6.48240	17.61810	H24	1.72930	2.49510	18.88810	H39	2.93010	7.65240	21.59640
H10	3.75590	7.02760	18.43460	H25	0.47670	3.75110	19.05700	H40	1.59080	6.47380	21.37950
C11	4.17580	8.33200	16.18890	C26	0.59720	7.56210	19.07320	C41	0.57880	8.83940	21.13310
H12	5.02440	8.37120	16.87190	C27	0.76350	6.42560	18.28110	H42	1.19470	9.29260	21.91450
C13	4.15050	9.16710	15.07030	H28	5.19870	5.39560	17.61600	H43	-0.00610	9.60920	20.62470
H14	4.97620	9.85150	14.88530	H29	4.19800	5.11680	16.17300	H44	-0.08630	8.08340	21.55340
C15	2.16790	4.35340	17.87400	H30	3.03940	9.77270	13.31970				

syn-(2*S*,3*S*)-TSadd

E(B3LYP-D3/6-31G*(dichloromethane)) = -1229.62406820770
E(B3LYP-D3/6-311+G**(dichloromethane)) = -1229.95865196375
G_{tot}(B3LYP-D3/6-31G*(dichloromethane)) = -1229.179525

Number of imaginary frequencies: 1 (-86.01)

C1	3.63970	10.38470	14.83120	H2	3.72710	10.12350	13.77950	O3	0.54780	6.49100	18.73070

N4	2.48260	7.51430	18.04360	H23	3.71930	5.83460	19.81910	H42	3.65770	12.51410	14.46870
C5	3.59720	11.72750	15.21780	H24	3.92300	4.45410	18.71630	C43	-1.10230	8.97680	17.20240
C6	3.55730	9.37420	15.78670	H25	2.29100	4.95380	19.24830	O44	-1.88830	9.90470	17.53670
H7	3.58920	8.33280	15.48390	C26	0.56830	8.86530	18.70310	H45	-1.31290	7.93650	17.51020
C8	3.43970	9.69110	17.14550	C27	1.18410	7.55550	18.52940	C46	0.83780	9.10730	13.36830
C9	3.46730	8.60560	18.20820	N28	-0.11790	9.08040	20.04560	C47	0.76000	7.90320	14.07350
H10	3.34030	9.07310	19.19110	H29	1.18810	9.73250	18.51150	C48	0.17690	7.87480	15.34050
C11	3.39750	11.03700	17.52610	H30	-2.13490	8.77320	19.47320	C49	-0.33380	9.04500	15.91650
H12	3.31430	11.29480	18.58140	H31	-1.24070	7.28890	19.95490	C50	-0.24290	10.24950	15.20830
C13	3.47120	12.05280	16.56890	H32	-1.78970	8.46100	21.20430	C51	0.34120	10.28180	13.94310
H14	3.43430	13.09490	16.88040	C33	-1.43090	8.33600	20.17960	H52	1.28500	9.13130	12.37650
C15	3.18300	6.22900	17.73920	C34	-0.39780	10.55740	20.17540	H53	1.14370	6.98610	13.63100
O16	4.49430	6.67290	17.36660	H35	-0.96410	10.72920	21.09420	H54	0.09760	6.93870	15.88670
C17	4.79200	7.80880	18.17560	H36	0.55440	11.09090	20.22050	H55	-0.65920	11.14720	15.65710
C18	2.58940	5.50740	16.53630	H37	-0.97180	10.86410	19.30170	H56	0.40650	11.22160	13.39890
H19	3.21450	4.64350	16.28390	C38	0.77720	8.65710	21.18660	H57	5.09850	7.50920	19.18760
H20	2.54570	6.18070	15.67700	H39	0.29830	8.90570	22.13880	H58	5.61090	8.35710	17.70580
H21	1.58160	5.16060	16.77500	H40	0.93490	7.58050	21.11530				
C22	3.28230	5.30730	18.96470	H41	1.72680	9.18790	21.09580				

anti-(2*S*,3*S*)-TSadd

E(B3LYP-D3/6-31G*(dichloromethane)) = -1229.60991115648
E(B3LYP-D3/6-311+G**(dichloromethane)) = -1229.94586536873
G_{tot}(B3LYP-D3/6-31G*(dichloromethane)) = -1229.164499

Number of imaginary frequencies: 2 (-193.93, -13.23)

C1	2.93550	9.77290	14.71220	H21	1.88800	4.74140	17.71660	H41	1.34670	9.69060	20.87360
H2	2.85210	9.33750	13.71860	C22	3.67710	5.48980	19.64980	H42	5.26940	7.87760	19.30490
O3	0.75670	6.34510	19.28840	H23	4.16770	6.18370	20.34030	H43	5.55750	8.47680	17.65050
N4	2.59610	7.40660	18.45080	H24	4.33060	4.62350	19.50030	H44	2.71480	11.80530	14.01290
C5	2.85570	11.15950	14.87740	H25	2.73540	5.16580	20.09550	C45	-0.12710	8.35320	16.71220
C6	3.10880	8.93910	15.81670	C26	0.50280	8.62030	18.58830	O46	0.57480	7.45930	16.13990
H7	3.14950	7.86460	15.69100	C27	1.28250	7.38500	18.84130	H47	0.06420	9.42050	16.45750
C8	3.20480	9.48430	17.10210	N28	-0.36940	9.02840	19.80000	C48	-4.35550	7.50330	17.03620
C9	3.44970	8.60590	18.31810	H29	1.12940	9.49290	18.43500	C49	-3.93190	8.81200	16.79180
H10	3.35620	9.22400	19.21960	H30	-2.27510	8.24750	19.26510	C50	-2.56510	9.09530	16.68770
C11	3.12850	10.87330	17.26150	H31	-1.18150	7.09670	20.07860	C51	-1.61240	8.08090	16.83770
H12	3.20440	11.30480	18.25960	H32	-1.98760	8.40400	21.02160	C52	-2.04840	6.76400	17.04140
C13	2.95550	11.71110	16.15630	C33	-1.54890	8.11950	20.06150	C53	-3.40710	6.47750	17.15330
H14	2.89490	12.78880	16.29560	C34	-0.88500	10.41990	19.55090	H54	-5.41710	7.27910	17.12010
C15	3.40040	6.16120	18.29730	H35	-1.53340	10.71320	20.38120	H55	-4.66340	9.60920	16.67250
O16	4.62300	6.65490	17.73350	H36	-0.03970	11.10840	19.47930	H56	-2.23610	10.11280	16.47460
C17	4.85000	7.94610	18.29100	H37	-1.44710	10.41830	18.61870	H57	-1.29300	5.98830	17.12940
C18	2.78190	5.19940	17.29000	C38	0.47320	9.06130	21.05620	H58	-3.73500	5.45470	17.33100
H19	3.51000	4.41850	17.04790	H39	-0.11690	9.47430	21.87990				
H20	2.49020	5.74080	16.38910	H40	0.77990	8.04200	21.28930				

syn-(2*R*,3*S*)-TSadd

E(B3LYP-D3/6-31G*(dichloromethane)) = -1229.62788596370
E(B3LYP-D3/6-311+G**(dichloromethane)) = -1229.96383533691
G_{tot}(B3LYP-D3/6-31G*(dichloromethane)) = -1229.183719

Number of imaginary frequencies: 1 (-211.80)

C1	3.56930	8.13410	14.53890	C6	3.47490	7.37520	15.70580	C11	3.67390	9.38790	17.02840
H2	3.53460	7.63990	13.57000	H7	3.37850	6.29520	15.64750	H12	3.69950	9.87510	18.00300
O3	-0.10890	6.27370	18.79340	C8	3.52060	7.99630	16.96120	C13	3.76570	10.14980	15.86000
N4	2.13110	6.46100	18.39510	C9	3.40990	7.19630	18.24930	H14	3.87840	11.22990	15.92710
C5	3.71030	9.52430	14.61240	H10	3.55860	7.87560	19.09060	C15	2.34100	4.98970	18.22890

C16	4.40440	6.02590	18.35980	H31	0.22410	10.86630	19.57420	H46	-1.17270	6.26010	23.15950
C17	0.87760	8.46780	18.90900	H32	0.46620	10.93030	17.81460	H47	0.57190	6.60770	21.43270
C18	0.89910	7.00260	18.67860	H33	4.66980	5.84380	19.41080	H48	0.52420	10.82410	22.18520
N19	-0.44050	9.13930	18.52470	H34	5.31830	6.17980	17.78230	H49	-1.25930	10.49890	23.91520
H20	1.61640	8.98850	18.31360	H35	3.77990	10.11480	13.70130	O50	3.70670	4.91210	17.80350
H21	-1.67580	7.69500	19.45930	C36	1.71280	8.97360	20.63330	C51	2.15100	4.25620	19.56130
H22	-1.44840	9.19850	20.39820	O37	2.25220	10.12850	20.51290	H52	2.37180	3.19120	19.43010
H23	-2.51350	9.20740	18.96340	H38	2.37270	8.07780	20.61400	H53	2.81910	4.66390	20.32670
C24	-1.61320	8.77850	19.41010	C39	-1.34200	8.36010	23.63770	H54	1.11800	4.36920	19.89870
C25	-0.80720	8.79270	17.09690	C40	-0.81260	7.26440	22.94530	C55	1.49930	4.37760	17.11110
H26	-1.06790	7.73570	17.05570	C41	0.17470	7.45880	21.98050	H56	1.85470	3.35950	16.91740
H27	-1.65580	9.40960	16.78670	C42	0.63960	8.74830	21.68430	H57	0.44850	4.34680	17.40030
H28	0.05680	8.99690	16.46180	C43	0.12780	9.83530	22.40070	H58	1.60040	4.96780	16.19610
C29	-0.22230	10.63250	18.60850	C44	-0.86420	9.64550	23.36710				
H30	-1.18470	11.13150	18.47320	H45	-2.11380	8.20890	24.38970				

anti-(2*R*,3*S*)-TSadd

E(B3LYP-D3/6-31G*(dichloromethane)) = -1229.62606555654

E(B3LYP-D3/6-311+G**(dichloromethane)) = -1229.96307633507

G_{tot}(B3LYP-D3/6-31G*(dichloromethane)) = -1229.183450

Number of imaginary frequencies: 1 (-235.47)

C1	2.64040	7.94510	14.50880	H21	-1.26110	8.31720	20.15630	C41	1.29850	11.40030	21.68590
H2	2.16600	7.42520	13.67880	H22	-0.80050	9.97350	20.65450	C42	2.16300	10.55510	20.98040
O3	0.18800	6.61770	19.57580	H23	-2.08810	9.74480	19.43590	C43	3.24310	11.12480	20.29150
N4	2.26570	6.62230	18.63130	C24	-1.14910	9.35010	19.83350	C44	3.42070	12.50620	20.25360
C5	3.12240	9.24700	14.33530	C25	-0.67500	8.68040	17.51610	H45	2.66240	14.42690	20.90030
C6	2.76490	7.30890	15.74500	H26	-0.97370	7.67280	17.79740	H46	0.79110	13.43280	22.20840
H7	2.39110	6.29790	15.87570	H27	-1.53660	9.24540	17.14950	H47	0.48310	10.97140	22.26750
C8	3.37070	7.96270	16.82690	H28	0.10790	8.64490	16.75590	H48	3.93990	10.46050	19.78660
C9	3.50790	7.28830	18.18330	C29	0.14140	10.81350	18.34290	H49	4.25490	12.93250	19.69950
H10	3.81970	8.00980	18.94050	H30	-0.81010	11.27140	18.06070	O50	3.66450	4.94790	17.97530
C11	3.86050	9.26160	16.64050	H31	0.58200	11.34300	19.18340	C51	1.36710	4.35580	17.97920
H12	4.33710	9.77850	17.47140	H32	0.82620	10.82570	17.49260	H52	1.65980	3.30080	17.94230
C13	3.73720	9.90280	15.40410	H33	5.01600	6.03470	19.14620	H53	0.41390	4.45220	18.49940
H14	4.11760	10.91440	15.27830	H34	5.22060	6.14020	17.37670	H54	1.25370	4.72340	16.95500
C15	2.45100	5.14440	18.70930	H35	3.01950	9.74480	13.37340	C55	2.60830	4.70510	20.16940
C16	4.48760	6.09490	18.18540	C36	1.97890	9.04440	20.96260	H56	2.88700	3.64650	20.20670
C17	1.21770	8.74250	19.18640	O37	3.03400	8.32200	21.07360	H57	3.37170	5.30590	20.67160
C18	1.16830	7.25480	19.13550	H38	1.07420	8.75170	21.54230	H58	1.66300	4.85230	20.69720
N19	-0.11230	9.37980	18.73850	C39	2.52740	13.34790	20.92990				
H20	1.95940	9.17980	18.52850	C40	1.47460	12.78920	21.65810				

syn-(2*R*,3*R*)-TSadd

E(B3LYP-D3/6-31G*(dichloromethane)) = -1229.63368271840

E(B3LYP-D3/6-311+G**(dichloromethane)) = -1229.96738708511

G_{tot}(B3LYP-D3/6-31G*(dichloromethane)) = -1229.188161

Number of imaginary frequencies: 1 (-42.32)

C1	0.62060	7.99310	13.52680	H10	0.82100	10.70040	17.18180	H19	5.77840	10.45480	15.98140
H2	1.11290	7.27390	12.87510	C11	-0.62820	9.83010	15.20870	H20	4.42630	9.82700	15.00900
O3	3.58530	7.73020	17.98190	H12	-1.11680	10.53950	15.87410	H21	5.12220	8.81840	16.29560
N4	2.62520	9.60580	17.05370	C13	-1.36510	9.18800	14.20910	C22	4.50340	10.58280	18.40860
C5	-0.74230	8.26630	13.36490	H14	-2.42510	9.40550	14.09300	H23	3.76650	11.01310	19.09190
C6	1.35340	8.63940	14.52330	C15	3.87190	10.40450	17.03130	H24	5.35720	11.26570	18.33270
H7	2.40820	8.41580	14.65960	O16	3.42010	11.70800	16.62040	H25	4.83710	9.61860	18.79390
C8	0.73570	9.56480	15.37390	C17	2.27390	11.52480	15.80110	C26	2.51930	8.36550	17.70340
C9	1.52110	10.33010	16.42630	C18	4.86970	9.84240	16.01040	H27	1.68590	12.44690	15.81050

H28	2.55670	11.29660	14.76270	C39	1.54100	5.62260	17.13550	C50	-0.09690	10.44580	19.79280
H29	-1.31350	7.76240	12.58780	H40	2.61350	5.80380	17.08500	C51	-1.33690	10.67180	19.17490
C30	1.21360	7.93200	18.02210	H41	1.32940	4.56640	17.32950	C52	-1.57690	11.87440	18.51500
H31	0.34400	8.38220	17.57260	H42	1.05950	5.94810	16.21200	H53	-0.77510	13.80350	17.96230
N32	0.98920	6.46850	18.26160	C43	-0.49380	6.23240	18.35040	H54	1.40820	13.42590	19.09250
C33	0.17860	9.17260	20.49540	H44	-0.67250	5.17020	18.53620	H55	1.83920	11.27070	20.25930
H34	1.22650	9.03890	20.82100	H45	-0.88450	6.83340	19.17180	H56	-2.09220	9.89120	19.21100
C35	1.61650	6.02980	19.56430	H46	-0.95240	6.52790	17.40420	H57	-2.53320	12.04640	18.02600
H36	1.10950	6.55970	20.37020	C47	-0.58630	12.86640	18.48180	O58	-0.68520	8.34930	20.79840
H37	1.48750	4.94930	19.67910	C48	0.64150	12.65570	19.11670				
H38	2.66980	6.30000	19.51940	C49	0.88500	11.44640	19.76840				

anti-(2*R*,3*R*)-TSadd

E(B3LYP-D3/6-31G*(dichloromethane)) = -1229.62445667172

E(B3LYP-D3/6-311+G**(dichloromethane)) = -1229.96152853826

G_{tot}(B3LYP-D3/6-31G*(dichloromethane)) = -1229.179587

Number of imaginary frequencies: 2 (-220.54, -10.63)

C1	1.11890	8.39210	13.11140	H21	5.37990	8.69900	16.89140	H41	1.26190	4.96950	16.96010
H2	1.73810	7.99320	12.31080	C22	4.47350	11.04300	17.99060	H42	0.97730	6.52650	16.11650
O3	3.53470	8.06180	18.33300	H23	3.70820	11.71980	18.38100	C43	-0.48250	6.34770	18.37730
N4	2.66060	9.67500	16.98770	H24	5.37890	11.61370	17.75940	H44	-0.61250	5.26280	18.40100
C5	-0.26290	8.17340	13.10340	H25	4.70350	10.30000	18.75750	H45	-0.85200	6.78670	19.30260
C6	1.70770	9.12110	14.14390	C26	2.52990	8.61600	17.83900	H46	-1.01950	6.76790	17.52460
H7	2.77860	9.29810	14.14270	H27	2.22410	12.46040	16.69000	C47	2.09830	7.91510	23.27920
C8	0.92740	9.64190	15.18590	H28	1.92120	12.11210	14.96620	C48	0.75890	7.66790	22.96690
C9	1.55350	10.41460	16.33640	H29	-0.72080	7.59850	12.30180	C49	0.22450	8.14000	21.76290
H10	0.80290	10.66180	17.08900	C30	1.13640	8.19520	18.16760	C50	1.01810	8.85700	20.85910
C11	-0.45720	9.43340	15.15960	H31	0.43600	8.46960	17.38660	C51	2.34800	9.13600	21.20000
H12	-1.07610	9.84590	15.95490	N32	0.98480	6.66240	18.24060	C52	2.88870	8.65860	22.39310
C13	-1.05120	8.70160	14.12730	C33	0.43690	9.37380	19.55700	H53	2.52140	7.54400	24.21010
H14	-2.12740	8.54280	14.12640	H34	-0.61360	9.01200	19.45030	H54	0.12950	7.11270	23.66010
C15	3.97280	10.33600	16.72630	C35	1.71650	6.00720	19.39180	H55	-0.82230	7.95040	21.52360
O16	3.64660	11.28470	15.70430	H36	1.29900	6.37430	20.32420	H56	2.95070	9.71560	20.50870
C17	2.29260	11.69530	15.90500	H37	1.57540	4.92690	19.31000	H57	3.92920	8.86450	22.63770
C18	5.02740	9.40150	16.13580	H38	2.76370	6.28150	19.32040	O58	0.69090	10.58840	19.22470
H19	5.86840	10.00310	15.77340	C39	1.48270	6.04070	16.95290				
H20	4.61050	8.83830	15.29570	H40	2.55790	6.20190	16.89020				

syn-(2*S*,3*R*)-TSadd

E(B3LYP-D3/6-31G*(dichloromethane)) = -1229.62604962736

E(B3LYP-D3/6-311+G**(dichloromethane)) = -1229.96116786361

G_{tot}(B3LYP-D3/6-31G*(dichloromethane)) = -1229.180327

Number of imaginary frequencies: 1 (-114.78)

C1	3.42530	9.09000	14.48300	H14	3.81060	12.13940	15.94530	C27	1.31870	7.56550	18.96310
H2	3.29400	8.62830	13.50650	C15	2.80220	5.71750	18.10960	N28	0.43090	9.34150	20.49600
O3	0.45740	6.72090	19.28470	O16	4.10130	5.77410	17.50630	H29	2.01430	9.60920	19.14630
N4	2.48880	7.16390	18.34600	C17	4.78070	6.90040	18.05650	H30	0.76240	7.49380	21.49480
C5	3.53810	10.47990	14.59330	C18	1.86470	5.05790	17.10470	H31	2.09320	8.68370	21.64490
C6	3.47950	8.29000	15.62330	H19	2.19110	4.02670	16.92860	H32	0.58170	8.91000	22.57820
H7	3.40560	7.21040	15.53430	H20	1.88300	5.59840	16.15540	C33	1.00980	8.54330	21.64080
C8	3.63970	8.86560	16.89260	H21	0.84530	5.05710	17.49270	C34	-1.06100	9.10350	20.43980
C9	3.69490	7.99380	18.13720	C22	2.87730	4.92920	19.42490	H35	-1.21790	8.07930	20.10940
H10	3.87370	8.62630	19.01340	H23	3.57430	5.40280	20.12400	H36	-1.47450	9.26710	21.43880
C11	3.77140	10.25410	16.99210	H24	3.22970	3.91400	19.21310	H37	-1.47100	9.81570	19.72730
H12	3.90960	10.71520	17.96890	H25	1.89230	4.88400	19.89100	C38	0.67400	10.80540	20.78460
C13	3.71790	11.05970	15.85010	C26	1.10520	9.01630	19.16120	H39	0.09860	11.08380	21.67120

H40	1.74080	10.94780	20.97460	H47	1.03810	10.06580	17.08090	H54	-2.93340	6.09570	15.30660
H41	0.35030	11.36790	19.90830	C48	-2.32600	6.86410	15.78080	H55	-0.46280	6.11420	14.98790
H42	5.18440	6.67550	19.05380	C49	-0.93920	6.87430	15.60400	H56	0.91220	7.87040	16.06950
H43	5.60520	7.16140	17.38970	C50	-0.16420	7.86050	16.21350	H57	-2.61050	9.64380	17.73410
H44	3.48780	11.10490	13.70440	C51	-0.75440	8.84630	17.01440	H58	-4.00900	7.86980	16.68460
C45	0.08550	9.94920	17.62820	C52	-2.14690	8.84620	17.16040				
O46	-0.50030	11.00140	18.06290	C53	-2.92760	7.85840	16.55820				

anti-(2*S*,3*R*)-TSadd

E(B3LYP-D3/6-31G*(dichloromethane)) = -1229.61507851980

E(B3LYP-D3/6-311+G**(dichloromethane)) = -1229.95252247799

G_{tot}(B3LYP-D3/6-31G*(dichloromethane)) = -1229.170880

Number of imaginary frequencies: 1 (-192.33)

C1	3.61090	8.75290	14.19450	H21	1.37560	4.58390	17.89930	H41	-0.33130	11.17520	18.04140
H2	3.42610	8.20270	13.27450	C22	3.31610	5.17040	19.67740	H42	5.37570	7.07120	19.07770
O3	0.68030	6.67830	19.26270	H23	3.99330	5.78620	20.27820	H43	5.80100	7.38120	17.37320
N4	2.68100	7.15020	18.26680	H24	3.72630	4.15620	19.61890	H44	3.99680	10.62420	13.18340
C5	3.92770	10.11500	14.14240	H25	2.34230	5.14380	20.16850	C45	-0.18900	8.26380	16.74730
C6	3.51370	8.09640	15.42110	C26	0.89100	8.80010	18.19410	O46	0.46900	7.45230	15.99320
H7	3.25040	7.04660	15.45940	C27	1.41350	7.48080	18.65260	H47	-1.03080	7.84630	17.35110
C8	3.74740	8.79290	16.61250	N28	0.28770	9.62190	19.35730	C48	-1.29950	12.03370	14.90470
C9	3.78290	8.08530	17.95620	H29	1.64360	9.44490	17.75190	C49	-2.25440	11.31100	15.62320
H10	3.85400	8.84110	18.74750	H30	1.12880	8.49220	20.95470	C50	-1.90830	10.09080	16.21810
C11	4.05680	10.15730	16.55550	H31	2.16950	9.81240	20.33390	C51	-0.60790	9.58470	16.10800
H12	4.23980	10.70710	17.47860	H32	0.75770	10.19440	21.36370	C52	0.33290	10.30050	15.35250
C13	4.14550	10.81970	15.32790	C33	1.15050	9.52060	20.59680	C53	-0.00340	11.51830	14.76580
H14	4.38400	11.88100	15.29980	C34	-1.10660	9.16740	19.71450	H54	-1.56350	12.98470	14.44590
C15	3.16290	5.73710	18.26000	H35	-1.07200	8.09460	19.89510	H55	-3.26980	11.69230	15.71700
O16	4.44990	5.86220	17.64040	H36	-1.43010	9.71100	20.60610	H56	-2.66250	9.52370	16.76400
C17	4.98670	7.11700	18.05070	H37	-1.76860	9.39290	18.88130	H57	1.32840	9.88400	15.23740
C18	2.29290	4.85350	17.37160	C38	0.24010	11.07700	18.96130	H58	0.74340	12.06920	14.19650
H19	2.84730	3.94320	17.12050	H39	-0.23230	11.64390	19.76780				
H20	2.01150	5.39410	16.46560	H40	1.26080	11.43320	18.80670				

syn-(2*S*,3*S*)-Betaine

E(B3LYP-D3/6-31G*(dichloromethane)) = -1229.63365904187

E(B3LYP-D3/6-311+G**(dichloromethane)) = -1229.97114088770

G_{tot}(B3LYP-D3/6-31G*(dichloromethane)) = -1229.186461

Number of imaginary frequencies: 0

C1	3.83650	10.56070	15.05560	C18	1.15960	7.57880	18.48090	O35	-1.76420	9.96410	18.06800
H2	4.08860	10.29410	14.03150	N19	-0.11520	8.88650	20.22390	H36	-1.32240	7.96740	17.74780
O3	0.55840	6.49260	18.48040	H20	0.98550	9.75520	18.64010	C37	0.77620	9.52550	13.73690
N4	2.49430	7.66280	18.21630	H21	-2.16170	8.60390	19.81980	C38	0.61620	8.23790	14.25190
C5	3.63710	11.90060	15.40260	H22	-1.21730	7.08380	20.04050	C39	0.09350	8.05820	15.53560
C6	3.71180	9.55930	16.01600	H23	-1.63820	8.09000	21.46830	C40	-0.27300	9.15790	16.32140
H7	3.88730	8.52380	15.74460	C24	-1.39330	8.09580	20.40350	C41	-0.11020	10.44210	15.79430
C8	3.38780	9.88390	17.34030	C25	-0.37640	10.33290	20.58780	C42	0.41320	10.62850	14.51500
C9	3.38400	8.82940	18.43390	H26	-0.85360	10.36090	21.57100	H43	1.18220	9.66960	12.73740
H10	3.14360	9.30910	19.38700	H27	0.58240	10.85700	20.62340	H44	0.89020	7.37230	13.65130
C11	3.18370	11.22660	17.67950	H28	-1.02220	10.72460	19.79680	H45	-0.03580	7.05390	15.93250
H12	2.94550	11.49330	18.70890	C29	0.92290	8.33440	21.16400	H46	-0.42470	11.28320	16.40520
C13	3.30510	12.23210	16.71670	H30	0.57560	8.48440	22.18960	H47	0.54180	11.63450	14.12180
H14	3.14600	13.27160	16.99630	H31	1.05410	7.26920	20.97150	O48	4.61130	6.99400	17.66550
C15	3.30490	6.44550	17.86100	H32	1.86180	8.87220	21.02040	H49	4.93120	7.77800	19.58050
C16	4.74360	8.10590	18.54800	H33	3.73770	12.68000	14.65030	H50	5.58260	8.71590	18.20820
C17	0.37130	8.86190	18.74430	C34	-0.89780	8.99860	17.72550	C51	2.86530	5.81890	16.54210

H52	3.61240	5.07970	16.23460	C55	3.33700	5.42450	19.00540	H58	2.34030	5.01730	19.18030
H53	1.90050	5.32300	16.66360	H56	4.01780	4.61180	18.73020				
H54	2.77450	6.58310	15.76640	H57	3.69920	5.88360	19.93090				

syn-(2*R*,3*S*)-Betaine

E(B3LYP-D3/6-31G*(dichloromethane)) = -1229.63074278585

E(B3LYP-D3/6-311+G**(dichloromethane)) = -1229.96993774571

G_{tot}(B3LYP-D3/6-31G*(dichloromethane)) = -1229.185987

Number of imaginary frequencies: 0

C1	3.33470	7.26350	13.83640	N21	-0.58930	8.81710	17.19520	H41	5.28740	7.55300	21.59430
H2	3.22760	6.67430	12.92770	C22	0.30570	8.73460	19.72810	H42	3.38490	6.00000	21.99730
O3	-0.52100	6.05830	18.18530	C23	-1.99690	8.63520	17.72460	H43	1.08980	6.60520	21.18210
N4	1.72290	6.10950	17.80250	H24	-2.01710	8.98120	18.75720	H44	2.61460	10.27300	19.59360
C5	3.70810	8.61070	13.75950	H25	-2.67160	9.19510	17.07250	H45	4.89000	9.71150	20.41630
C6	3.10790	6.66570	15.07690	H26	-2.22170	7.57170	17.71300	O46	-0.72040	8.23250	20.38370
H7	2.84570	5.61370	15.13120	C27	-0.48250	8.17780	15.82920	H47	0.28980	9.85110	19.58080
C8	3.24590	7.40400	16.26230	H28	-0.75920	7.12790	15.90990	O48	3.22630	4.43340	17.39240
C9	3.04390	6.75770	17.62570	H29	-1.16390	8.69190	15.14630	H49	4.20640	5.45620	18.93460
H10	3.20600	7.49660	18.40920	H30	0.54690	8.27270	15.47800	H50	4.90510	5.60690	17.29740
C11	3.63020	8.74890	16.17690	C31	-0.32300	10.29390	17.01570	C51	0.99670	3.92340	16.75320
H12	3.76520	9.32740	17.08950	H32	-1.05240	10.69420	16.30850	H52	1.32170	2.88130	16.66170
C13	3.85950	9.35090	14.93440	H33	-0.42020	10.80120	17.97450	H53	-0.05210	3.95200	17.04950
H14	4.16200	10.39500	14.88720	H34	0.68650	10.42550	16.61990	H54	1.10610	4.41310	15.78130
C15	1.86630	4.61480	17.80000	C35	4.28920	7.80910	21.24540	C55	1.64530	4.04460	19.20490
C16	3.97410	5.55230	17.86590	C36	3.21840	6.93630	21.46840	H56	1.80560	2.96130	19.18430
C17	0.51930	6.71410	18.04160	C37	1.93770	7.26380	21.01550	H57	2.34200	4.49340	19.91830
H18	3.88730	9.07460	12.79200	C38	1.71130	8.45170	20.31630	H58	0.62510	4.25250	19.53570
C19	0.49250	8.23290	18.14370	C39	2.78350	9.33410	20.12200				
H20	1.40940	8.67090	17.75450	C40	4.06590	9.01950	20.58110				

syn-(2*R*,3*R*)-Betaine

E(B3LYP-D3/6-31G*(dichloromethane)) = -1229.64146288472

E(B3LYP-D3/6-311+G**(dichloromethane)) = -1229.97766697531

G_{tot}(B3LYP-D3/6-31G*(dichloromethane)) = -1229.193229

Number of imaginary frequencies: 0

C1	1.12590	7.96260	13.18760	N21	0.86870	6.68200	18.31280	C41	-1.38020	11.98910	18.52730
H2	1.78510	7.52960	12.43730	C22	0.32560	8.80740	19.80060	H42	-0.52440	13.95930	18.27170
O3	3.24050	8.36630	18.74980	H23	1.24020	8.76040	20.44110	H43	1.67450	13.31400	19.24360
N4	2.44700	9.65340	17.04890	C24	1.35810	6.07050	19.60590	H44	2.06690	10.97720	19.98760
C5	-0.24720	7.69440	13.15510	H25	0.65510	6.37590	20.38140	H45	-1.93820	9.92630	18.91330
C6	1.65570	8.79140	14.17730	H26	1.37130	4.98480	19.48190	H46	-2.34950	12.27060	18.11950
H7	2.71790	9.01530	14.18530	H27	2.35900	6.45190	19.79970	O47	-0.73560	8.15850	20.28890
C8	0.82200	9.36220	15.15190	C28	1.70080	6.16320	17.16750	O48	3.49960	11.01900	15.54770
C9	1.38290	10.27810	16.22780	H29	2.75490	6.33140	17.38490	H49	2.08510	12.34060	16.34700
H10	0.57690	10.63050	16.87290	H30	1.50720	5.09300	17.05870	H50	1.81360	11.78940	14.66930
C11	-0.55290	9.09940	15.10460	H31	1.41150	6.69050	16.25610	C51	4.81600	9.18850	16.30830
H12	-1.21240	9.54950	15.84530	C32	-0.56840	6.26000	18.08260	H52	5.68750	9.69390	15.87860
C13	-1.08660	8.26860	14.11300	H33	-0.62820	5.17310	18.17980	H53	5.12830	8.60810	17.17740
H14	-2.15670	8.07380	14.08990	H34	-1.15880	6.77850	18.84190	H54	4.39580	8.51220	15.55870
C15	3.78900	10.24280	16.71350	H35	-0.85430	6.56230	17.07230	C55	4.30360	11.14420	17.84060
C16	2.15520	11.48610	15.66120	C36	-0.35600	12.94000	18.61330	H56	5.25550	11.59060	17.53310
C17	2.28450	8.75400	18.05720	C37	0.88030	12.57500	19.15390	H57	3.59160	11.94570	18.05420
H18	-0.65870	7.04720	12.38330	C38	1.09790	11.26110	19.58150	H58	4.45850	10.55810	18.74910
C19	0.88210	8.23010	18.35900	C39	0.09050	10.29660	19.46930				
H20	0.16890	8.50710	17.58790	C40	-1.15700	10.68090	18.96100				

syn-(2*S*,3*R*)-Betaine

E(B3LYP-D3/6-31G*(dichloromethane)) = -1229.62832739197
E(B3LYP-D3/6-311+G**(dichloromethane)) = -1229.96628557283
G_{tot}(B3LYP-D3/6-31G*(dichloromethane)) = -1229.181674

Number of imaginary frequencies: 0

C1	3.49650	9.10110	14.57310	H21	0.81410	7.34470	21.43720	C41	-2.11380	8.82550	17.31280
H2	3.38120	8.62780	13.60030	H22	2.18530	8.49690	21.50510	C42	-2.94790	7.92270	16.64790
O3	0.49130	6.69010	19.19700	H23	0.76850	8.73060	22.56710	H43	-3.04960	6.23710	15.29630
N4	2.57160	7.18030	18.41970	C24	1.10130	8.38320	21.58570	H44	-0.57780	6.13710	14.98460
C5	3.57470	10.49450	14.67210	C25	-1.04520	8.97710	20.53640	H45	0.88310	7.74670	16.16520
C6	3.56340	8.31280	15.72080	H26	-1.20640	7.98090	20.13100	H46	-2.53180	9.62090	17.92280
H7	3.51590	7.23110	15.64030	H27	-1.40230	9.04590	21.56710	H47	-4.02790	7.99350	16.76760
C8	3.70190	8.90550	16.98490	H28	-1.49390	9.74040	19.90460	O48	4.25320	5.84050	17.63380
C9	3.75870	8.05020	18.23910	C29	0.67950	10.69070	20.91710	H49	5.24740	6.77700	19.22010
H10	3.89190	8.69340	19.11520	H30	0.11610	10.89700	21.83100	H50	5.71860	7.27170	17.57110
C11	3.79880	10.29760	17.07400	H31	1.74900	10.82620	21.10010	C51	2.05980	5.05940	17.13560
H12	3.91580	10.77110	18.04780	H32	0.33100	11.28600	20.06880	H52	2.44420	4.04960	16.95410
C13	3.73290	11.09080	15.92420	H33	3.51280	11.11000	13.77750	H53	1.02900	4.99710	17.48690
H14	3.79610	12.17330	16.01010	C34	0.19300	9.77390	17.89120	H54	2.08320	5.61570	16.19550
C15	2.93590	5.73930	18.18310	O35	-0.40800	10.89870	18.27540	C55	2.97480	4.95110	19.49870
C16	4.87740	6.98740	18.20670	H36	1.04830	9.91520	17.18920	H56	3.35910	3.94530	19.29790
C17	1.06760	9.02410	19.13310	C37	-2.40200	6.93920	15.81840	H57	3.63150	5.43750	20.22740
C18	1.35430	7.53960	18.92990	C38	-1.01580	6.88160	15.64720	H58	1.97230	4.87680	19.92330
N19	0.44200	9.24330	20.53510	C39	-0.19220	7.78540	16.31920				
H20	1.98940	9.60010	19.18980	C40	-0.72440	8.74980	17.18460				

(2*S*,3*S*)-TSrot

E(B3LYP-D3/6-31G*(dichloromethane)) = -1229.60625143297
E(B3LYP-D3/6-311+G**(dichloromethane)) = -1229.94712201667
G_{tot}(B3LYP-D3/6-31G*(dichloromethane)) = -1229.159968

Number of imaginary frequencies: 1 (-29.73)

C1	2.44990	11.01050	15.59570	H21	1.73490	5.60010	16.56210	H41	1.78800	8.55730	21.23670
H2	1.96310	10.97630	14.62360	C22	2.86810	5.13930	19.14510	H42	5.61430	8.27430	18.29080
O3	0.40840	6.53770	18.61730	H23	2.99510	5.56890	20.14450	H43	4.73400	8.01290	16.76170
N4	2.44570	7.51410	18.34520	H24	3.60490	4.33940	19.01350	H44	2.80390	13.14050	15.52220
C5	2.91240	12.22770	16.10430	H25	1.86380	4.72890	19.05230	C45	-0.28720	9.42120	17.59480
C6	2.57890	9.83710	16.34010	C26	0.49100	8.92440	18.93960	O46	-0.29130	8.47620	16.65300
H7	2.12370	8.91650	15.98660	C27	1.10220	7.55940	18.59010	H47	0.36390	10.29810	17.35840
C8	3.17210	9.87520	17.60830	N28	-0.15300	8.79780	20.37800	C48	-4.20060	11.28420	18.11630
C9	3.43690	8.61270	18.40490	H29	1.27520	9.65280	19.14480	C49	-3.04030	12.03590	18.32060
H10	3.61370	8.87790	19.45690	H30	-2.21640	8.66270	19.89660	C50	-1.78670	11.43440	18.17170
C11	3.62690	11.09800	18.11910	H31	-1.32780	7.11440	19.87450	C51	-1.66660	10.07760	17.83810
H12	4.08570	11.13330	19.10660	H32	-1.74790	7.89330	21.44230	C52	-2.83560	9.34910	17.59330
C13	3.49850	12.27100	17.37170	C33	-1.46780	8.05630	20.39840	C53	-4.09260	9.93860	17.74330
H14	3.85150	13.21560	17.78070	C34	-0.35960	10.17600	20.95320	H54	-5.17960	11.74670	18.22740
C15	3.13550	6.20570	18.08430	H35	-0.80020	10.07520	21.94770	H55	-3.11060	13.09080	18.58230
O16	4.52270	6.53820	18.22670	H36	0.60560	10.68150	21.02800	H56	-0.88360	12.03140	18.30410
C17	4.68650	7.89620	17.85330	H37	-1.02930	10.73450	20.30530	H57	-2.72230	8.31690	17.27130
C18	2.81560	5.72810	16.66640	C38	0.80760	8.08040	21.30350	H58	-4.99260	9.35410	17.56000
H19	3.31710	4.77430	16.47070	H39	0.43060	8.15950	22.32630				
H20	3.14920	6.46270	15.92670	H40	0.86790	7.03430	21.01160				

(2*R*,3*S*)-TSrot

E(B3LYP-D3/6-31G*(dichloromethane)) = -1229.62391467500
E(B3LYP-D3/6-311+G**(dichloromethane)) = -1229.96302023541
G_{tot}(B3LYP-D3/6-31G*(dichloromethane)) = -1229.176315

Number of imaginary frequencies: 1 (-26.91)

C1	2.00120	4.66370	14.81300	N21	0.41270	9.40130	16.61320	H41	1.04090	14.15450	20.40490
H2	1.64230	4.10750	13.94940	C22	0.83350	8.80930	19.39790	H42	3.13220	12.85940	20.01140
O3	-1.87230	7.97770	17.69200	C23	-0.60400	10.51260	16.72150	H43	2.99850	10.38870	19.58490
N4	-0.50450	6.19460	18.06870	H24	-0.27640	11.22040	17.47690	H44	-1.26060	10.54910	19.89280
C5	3.23550	5.32130	14.75960	H25	-0.66290	11.00370	15.74660	H45	-1.15760	12.98470	20.37270
C6	1.22450	4.72120	15.97130	H26	-1.55950	10.07240	16.99460	O46	1.92860	8.15030	19.78730
H7	0.26960	4.20490	16.01330	C27	0.09190	8.59740	15.36750	H47	-0.11190	8.47790	19.90340
C8	1.66960	5.43900	17.09010	H28	-0.94940	8.28340	15.40970	O48	-0.89810	3.96130	18.30710
C9	0.81870	5.56790	18.34410	H29	0.25470	9.23120	14.49260	H49	0.24160	4.30650	20.03000
H10	1.34810	6.17670	19.08560	H30	0.75230	7.72980	15.32840	H50	1.03400	3.40110	18.71230
C11	2.91460	6.08220	17.03590	C31	1.77690	10.01290	16.41080	C51	-2.49660	5.11010	16.96980
H12	3.24780	6.65670	17.89900	H32	1.72800	10.69660	15.55950	H52	-3.14680	4.23360	17.06020
C13	3.69320	6.02590	15.87550	H33	2.05820	10.55340	17.31140	H53	-3.11070	6.00710	16.87720
H14	4.65260	6.53830	15.84280	H34	2.49510	9.21600	16.20870	H54	-1.88180	5.00340	16.07150
C15	-1.61080	5.19700	18.21030	C35	0.99330	13.08690	20.20050	C55	-2.42040	5.46200	19.48460
C16	0.35810	4.22770	18.94050	C36	2.16640	12.35850	19.97710	H56	-3.18600	4.68670	19.59560
C17	-0.73030	7.50990	17.83830	C37	2.10100	10.98650	19.72320	H57	-1.77360	5.44990	20.36770
H18	3.83530	5.28300	13.85270	C38	0.86920	10.32830	19.65840	H58	-2.90470	6.43930	19.41930
C19	0.50250	8.41170	17.83040	C39	-0.29790	11.05920	19.91460				
H20	1.39050	7.85750	17.55790	C40	-0.24130	12.42980	20.17920				

(2*R*,3*R*)-TSrot

E(B3LYP-D3/6-31G*(dichloromethane)) = -1229.62897366155
E(B3LYP-D3/6-311+G**(dichloromethane)) = -1229.96799274974
G_{tot}(B3LYP-D3/6-31G*(dichloromethane)) = -1229.183116

Number of imaginary frequencies: 0 (smallest frequency: 20.92)

C1	0.79320	4.23250	15.01750	N21	2.63840	8.12920	18.54330	C41	-1.22690	12.15160	18.28880
H2	1.22950	3.86100	14.09230	C22	0.08220	8.71400	19.38660	H42	-0.81040	14.09020	19.15440
O3	1.64900	9.90250	16.54290	H23	0.67730	8.52760	20.31930	H43	0.66280	13.15200	20.93470
N4	-0.02390	8.46090	16.01540	C24	3.20970	9.42880	19.05380	H44	1.09240	10.71780	21.03850
C5	0.82840	3.44580	16.17390	H25	2.74320	9.66210	20.00820	H45	-1.48870	10.10230	17.68060
C6	0.19840	5.49510	15.04440	H26	4.28800	9.30390	19.18220	H46	-1.88880	12.54710	17.51920
H7	0.16220	6.09860	14.14220	H27	2.98740	10.20250	18.32340	O47	-1.05440	8.01350	19.31470
C8	-0.36820	5.98840	16.22890	C28	3.44080	7.67840	17.33850	O48	-1.26580	8.18230	14.11910
C9	-0.99530	7.37210	16.30590	H29	3.48490	8.48990	16.61660	H49	-2.78060	8.38090	15.55170
H10	-1.39850	7.53950	17.31070	H30	4.44710	7.41320	17.67190	H50	-2.54400	6.75530	14.85470
C11	-0.34090	5.18990	17.38080	H31	2.94920	6.80550	16.90380	C51	0.93880	9.01010	13.75400
H12	-0.76470	5.58890	18.30150	C32	2.81950	7.06850	19.60560	H52	0.62090	9.22260	12.72740
C13	0.25750	3.92610	17.35490	H33	3.87810	7.02620	19.87270	H53	1.67890	9.75000	14.06390
H14	0.27900	3.31920	18.25780	H34	2.22610	7.32200	20.48300	H54	1.39240	8.01490	13.78460
C15	-0.28380	9.06570	14.66790	H35	2.49880	6.10710	19.20000	C55	-0.84560	10.48740	14.78540
C16	-2.03240	7.65310	15.20750	C36	-0.62310	13.01950	19.20610	H56	-0.96540	10.91050	13.78220
C17	0.93820	8.93060	16.84740	C37	0.20760	12.49290	20.19800	H57	-1.81940	10.48490	15.28280
H18	1.29780	2.46430	16.15220	C38	0.45520	11.11580	20.24880	H58	-0.16000	11.11500	15.35730
C19	1.11650	8.21070	18.19050	C39	-0.11570	10.24260	19.31840				
H20	0.85960	7.16130	18.08900	C40	-0.99180	10.78020	18.36510				

(2*S*,3*R*)-TSrot

E(B3LYP-D3/6-31G*(dichloromethane)) = -1229.60846392035
E(B3LYP-D3/6-311+G**(dichloromethane)) = -1229.94876347338
G_{tot}(B3LYP-D3/6-31G*(dichloromethane)) = -1229.163720

Number of imaginary frequencies: 0 (smallest frequency: 20.09)

C1	4.18630	7.84440	14.09740	H21	2.78960	4.67960	17.39680	H41	-0.90790	10.95620	18.96370
H2	3.90960	7.15130	13.30520	C22	2.54120	5.52890	20.07380	H42	5.42880	8.51810	19.24240
O3	0.51470	6.42500	18.25700	H23	2.32810	6.30210	20.81620	H43	5.66910	7.36360	17.91420
N4	2.66450	7.29450	18.22490	H24	3.19030	4.77930	20.54100	H44	5.34940	9.11710	12.79240
C5	4.99240	8.94880	13.80680	H25	1.61210	5.06700	19.74720	C45	0.10300	8.99820	16.94610
C6	3.72140	7.62790	15.39690	C26	0.65700	8.88560	18.37630	O46	1.02280	8.02840	16.46940
H7	3.06690	6.79200	15.61290	C27	1.24210	7.44280	18.01310	H47	-0.94100	8.65000	16.88030
C8	4.05670	8.51130	16.42560	N28	-0.25420	9.03530	19.59820	C48	0.44180	12.91400	15.13550
C9	3.67670	8.28920	17.88800	H29	1.44540	9.62340	18.53370	C49	-0.77560	12.49710	15.68160
H10	3.40490	9.26980	18.30680	H30	0.49400	7.35400	20.61780	C50	-0.89170	11.22150	16.24100
C11	4.86450	9.61850	16.12660	H31	1.45310	8.84830	20.85410	C51	0.20810	10.35300	16.27690
H12	5.12390	10.32110	16.91850	H32	-0.12100	8.66030	21.68950	C52	1.41840	10.77210	15.71160
C13	5.33050	9.84010	14.82860	C33	0.44730	8.42740	20.78540	C53	1.53540	12.04320	15.14690
H14	5.95270	10.70710	14.61510	C34	-1.57850	8.33110	19.41230	H54	0.53330	13.90440	14.69410
C15	3.29820	6.13860	18.89740	H35	-1.35670	7.32950	19.04280	H55	-1.63770	13.16080	15.66190
O16	4.47750	6.72000	19.49560	H36	-2.08760	8.30620	20.37900	H56	-1.85160	10.89510	16.64080
C17	4.93680	7.74950	18.63680	H37	-2.18030	8.88920	18.69430	H57	2.25680	10.08680	15.70340
C18	3.69620	5.05500	17.88300	C38	-0.49680	10.49860	19.86390	H58	2.48310	12.35090	14.70890
H19	4.21310	4.22440	18.37780	H39	-1.20170	10.59510	20.69430				
H20	4.35430	5.46600	17.11160	H40	0.45230	10.97580	20.12060				

anti-(2*S*,3*S*)-Betaine

E(B3LYP-D3/6-31G*(dichloromethane)) = -1229.61428337523
E(B3LYP-D3/6-311+G**(dichloromethane)) = -1229.95265417309
G_{tot}(B3LYP-D3/6-31G*(dichloromethane)) = -1229.170012

Number of imaginary frequencies: 0

C1	-1.83090	8.22050	24.27000	H21	0.96620	8.26360	16.62030	C41	0.48510	10.87970	16.74800
H2	-2.21990	7.55860	25.04070	H22	2.47680	8.09610	17.54100	C42	0.57590	11.14560	15.38340
O3	2.43600	9.88100	19.00980	H23	1.71970	6.64810	16.78930	H43	-0.30470	10.83710	13.43040
N4	1.90880	9.40810	21.17510	C24	1.54060	7.61270	17.27160	H44	-2.18140	9.45810	14.31450
C5	-2.60260	9.29230	23.80530	C25	-0.54460	6.66940	18.15980	H45	-2.33560	8.98820	16.74380
C6	-0.56090	7.99700	23.73990	H26	-0.30690	5.71950	17.67340	H46	1.20170	11.28800	17.45100
H7	0.03160	7.15680	24.10220	H27	-1.12940	6.48950	19.06430	H47	1.38430	11.76900	15.00540
C8	-0.03980	8.84180	22.74610	H28	-1.09680	7.30790	17.47540	O48	2.83360	10.10200	23.15050
C9	1.36440	8.53450	22.23420	C29	1.54410	6.39780	19.38290	H49	3.28620	8.05830	23.17650
H10	1.39990	7.49050	21.90740	H30	1.76770	5.50260	18.79540	H50	2.05710	8.62670	24.33880
C11	-0.81500	9.90250	22.27310	H31	2.47690	6.87740	19.68330	C51	2.58660	11.78790	21.47860
H12	-0.47540	10.50570	21.42890	H32	0.95270	6.11810	20.25630	H52	3.32670	12.44240	21.95180
C13	-2.09250	10.11970	22.80460	H33	-3.59520	9.46870	24.21460	H53	2.56790	11.97970	20.40400
H14	-2.69180	10.94140	22.41700	C34	-0.64310	9.82110	18.76700	H54	1.59760	11.99590	21.89390
C15	2.94900	10.33170	21.74300	O35	-0.47220	10.93520	19.47120	C55	4.36620	9.97440	21.27840
C16	2.44070	8.74610	23.32400	H36	-1.62480	9.29460	18.91820	H56	5.08220	10.59050	21.83360
C17	0.40180	8.64740	19.35800	C37	-0.37350	10.62270	14.49490	H57	4.58770	8.91920	21.47460
C18	1.67660	9.35100	19.82820	C38	-1.42020	9.84440	14.98960	H58	4.47460	10.15960	20.20920
N19	0.74550	7.35690	18.53250	C39	-1.49830	9.57180	16.36070				
H20	-0.13690	8.23500	20.21040	C40	-0.53500	10.05920	17.25220				

anti-(2*R*,3*S*)-Betaine

E(B3LYP-D3/6-31G*(dichloromethane)) = -1229.63215275372
E(B3LYP-D3/6-311+G**(dichloromethane)) = -1229.97168894620
G_{tot}(B3LYP-D3/6-31G*(dichloromethane)) = -1229.186678

Number of imaginary frequencies: 0

C1	3.02540	7.52210	13.81750	N21	-0.63360	8.75620	17.12460	H41	1.06340	14.29060	19.52840
H2	2.83150	6.93650	12.92110	C22	0.58010	8.86930	19.62760	H42	2.99400	12.82390	18.95010
O3	-0.35540	6.17070	18.53490	C23	-1.97840	8.72180	17.80360	H43	2.74560	10.34250	19.08260
N4	1.80720	6.26710	17.80780	H24	-2.01140	9.50920	18.55430	H44	-1.32360	10.79710	20.33740
C5	3.35620	8.87750	13.71140	H25	-2.74960	8.89570	17.04830	H45	-1.08700	13.26590	20.25140
C6	2.94240	6.91590	15.07180	H26	-2.09780	7.74450	18.26770	O46	1.63690	8.24360	20.18070
H7	2.69370	5.86200	15.14990	C27	-0.66740	7.82490	15.93290	H47	-0.41530	8.59890	20.06150
C8	3.18410	7.65340	16.24070	H28	-0.99680	6.84060	16.25980	O48	3.40090	4.68210	17.44300
C9	3.07880	7.00460	17.61310	H29	-1.36810	8.22940	15.19790	H49	4.41580	5.82190	18.87790
H10	3.16520	7.74950	18.40850	H30	0.33490	7.77000	15.50480	H50	4.98410	5.96110	17.19290
C11	3.53170	9.00510	16.12350	C31	-0.38430	10.14420	16.58590	C51	1.16790	3.97450	17.01040
H12	3.73430	9.58680	17.02070	H32	-1.21650	10.41780	15.93200	H52	1.48700	2.92710	17.04010
C13	3.61580	9.61620	14.86770	H33	-0.31040	10.84320	17.41460	H53	0.12910	4.04410	17.33890
H14	3.88190	10.66860	14.79490	H34	0.54820	10.13580	16.01810	H54	1.24360	4.33680	15.98070
C15	2.06160	4.79930	17.93040	C35	0.95990	13.20810	19.57230	C55	1.97210	4.34710	19.39280
C16	4.10030	5.86830	17.82730	C36	2.04350	12.38220	19.24500	H56	2.30720	3.30720	19.46800
C17	0.63590	6.83400	18.19420	C37	1.90530	10.99680	19.30140	H57	2.59660	4.97740	20.03280
H18	3.41460	9.35170	12.73390	C38	0.68480	10.40360	19.65600	H58	0.94100	4.42490	19.74360
C19	0.53830	8.36140	18.10670	C39	-0.38020	11.23760	20.01550				
H20	1.40250	8.78860	17.60960	C40	-0.24750	12.63180	19.97190				

anti-(2*R*,3*R*)-Betaine

E(B3LYP-D3/6-31G*(dichloromethane)) = -1229.62952736211
E(B3LYP-D3/6-311+G**(dichloromethane)) = -1229.96835615836
G_{tot}(B3LYP-D3/6-31G*(dichloromethane)) = -1229.184116

Number of imaginary frequencies: 0

C1	1.09860	8.56190	13.04500	N21	1.11030	6.44850	18.09150	C41	2.91440	10.10440	21.71090
H2	1.68120	8.24770	12.18110	C22	0.26250	8.80740	19.26330	H42	3.31730	9.44480	23.73170
O3	3.47500	7.98090	18.44310	H23	-0.55210	8.08380	19.52800	H43	1.48790	7.75480	23.71690
N4	2.69810	9.57820	17.03040	C24	1.62960	5.84990	19.37530	H44	0.09220	7.45400	21.69380
C5	-0.26850	8.27040	13.11170	H25	0.91870	6.05850	20.17120	H45	2.25650	10.56850	19.71000
C6	1.72070	9.25600	14.08400	H26	1.71930	4.76940	19.23090	H46	3.69580	10.86190	21.71600
H7	2.77950	9.49130	14.02490	H27	2.59400	6.30040	19.59410	O47	-0.16250	9.98510	18.78850
C8	0.98410	9.66540	15.20510	C28	1.93960	5.92310	16.93650	O48	3.76380	11.12430	15.73180
C9	1.63130	10.38320	16.37840	H29	2.99180	6.09660	17.14750	H49	2.45640	12.35930	16.80480
H10	0.87730	10.60480	17.14080	H30	1.74770	4.85250	16.82970	H50	2.08710	12.10970	15.07660
C11	-0.38810	9.38320	15.25830	H31	1.63980	6.44940	16.02780	C51	4.98950	9.11420	16.09100
H12	-0.94940	9.68970	16.13990	C32	-0.30170	5.95510	17.87060	H52	5.87590	9.63150	15.70870
C13	-1.01210	8.68700	14.21860	H33	-0.29650	4.86300	17.90530	H53	5.29690	8.37170	16.82890
H14	-2.07640	8.46740	14.27670	H34	-0.95030	6.34730	18.65220	H54	4.48960	8.60800	15.26040
C15	4.05460	10.13990	16.72790	H35	-0.64600	6.29530	16.89220	C55	4.67640	10.79610	17.96610
C16	2.44590	11.62550	15.98710	C36	2.70220	9.31030	22.84430	H56	5.65480	11.21050	17.70040
C17	2.51940	8.52850	17.86910	C37	1.68130	8.35910	22.83240	H57	4.04290	11.60800	18.33610
H18	-0.75000	7.72340	12.30370	C38	0.89940	8.18620	21.68460	H58	4.80010	10.05740	18.76050
C19	1.08310	8.01820	18.06970	C39	1.12550	8.94720	20.53390				
H20	0.48040	8.21930	17.19030	C40	2.12160	9.93210	20.57870				

anti-(2*S*,3*R*)-Betaine

E(B3LYP-D3/6-31G*(dichloromethane)) = -1229.62050868966
E(B3LYP-D3/6-311+G**(dichloromethane)) = -1229.96072700484
G_{tot}(B3LYP-D3/6-31G*(dichloromethane)) = -1229.175139

Number of imaginary frequencies: 0

C1	3.51810	9.41210	14.65410	H21	1.16420	8.06430	20.98810	C41	0.11910	10.77970	15.87980
H2	2.92490	9.17500	13.77270	H22	2.16940	9.49440	20.57560	C42	-0.27780	12.02770	15.40360
O3	0.65610	6.52860	18.94070	H23	0.80480	9.65810	21.70790	H43	-1.90660	13.43980	15.21500
N4	2.73100	7.32960	18.41180	C24	1.16620	9.14000	20.81550	H44	-3.54000	11.95890	16.37180
C5	4.53710	10.36260	14.57670	C25	-1.13200	8.93700	20.02660	H45	-2.82270	9.74310	17.22500
C6	3.23270	8.76200	15.86210	H26	-1.10060	7.84840	19.99130	H46	1.13390	10.42900	15.71600
H7	2.37360	8.09480	15.92680	H27	-1.37690	9.29010	21.03230	H47	0.43780	12.66290	14.88450
C8	3.97730	9.06670	17.00330	H28	-1.85740	9.32320	19.31290	O48	4.77320	6.30840	18.21890
C9	3.75100	8.39520	18.35390	C29	0.17820	10.96180	19.53860	H49	5.02450	7.52170	19.90850
H10	3.53300	9.16690	19.10050	H30	-0.24410	11.37030	20.46040	H50	5.92930	7.98310	18.44100
C11	4.98770	10.03980	16.92900	H31	1.19010	11.34630	19.39290	C51	2.93860	5.17710	17.18340
H12	5.55920	10.29730	17.82050	H32	-0.44600	11.22210	18.68710	H52	3.44570	4.20600	17.16890
C13	5.27100	10.68250	15.72490	H33	4.75480	10.86080	13.63410	H53	1.85840	5.02140	17.22280
H14	6.06160	11.43020	15.68130	C34	-0.29220	8.58630	17.09520	H54	3.18600	5.72850	16.27270
C15	3.39140	5.97990	18.39650	O35	0.30300	7.83540	16.16100	C55	3.21460	5.22510	19.71790
C16	4.98330	7.58510	18.81100	H36	-1.16580	8.09840	17.59260	H56	3.83140	4.32070	19.68610
C17	0.76470	8.83660	18.31180	C37	-1.59570	12.46580	15.58750	H57	3.54100	5.84120	20.56260
C18	1.38390	7.46390	18.58360	C38	-2.50860	11.63670	16.24060	H58	2.17040	4.95130	19.86740
N19	0.23880	9.45810	19.66450	C39	-2.09960	10.38760	16.72590				
H20	1.50810	9.55290	17.96680	C40	-0.77870	9.95070	16.57100				

(2*S*,3*S*)-TSelim

E(B3LYP-D3/6-31G*(dichloromethane)) = -1229.60855020817
E(B3LYP-D3/6-311+G**(dichloromethane)) = -1229.94288786020
G_{tot}(B3LYP-D3/6-31G*(dichloromethane)) = -1229.166456

Number of imaginary frequencies: 1 (-278.96)

C1	-1.80310	11.81830	17.81170	H21	-0.64610	6.19150	17.31790	H41	-4.45420	5.19380	20.08760
H2	-1.98870	12.05260	16.76540	C22	1.62550	6.41790	18.88420	H42	2.56660	9.12520	19.68010
O3	-1.31770	8.18300	21.41290	H23	2.27310	6.77950	19.68940	H43	1.99350	10.50090	18.69930
N4	-0.09240	8.12950	19.51320	H24	2.25040	6.07300	18.05340	H44	-3.27830	13.21180	18.55400
C5	-2.52700	12.46960	18.81550	H25	1.02940	5.59300	19.27930	C45	-1.69060	5.17000	21.06970
C6	-0.84110	10.86480	18.14780	C26	-1.67810	6.27300	20.04210	O46	-0.32670	5.05870	20.90710
H7	-0.28300	10.35670	17.36570	C27	-0.98830	7.57190	20.39080	H47	-2.27380	4.31340	20.67290
C8	-0.58800	10.55020	19.49160	N28	-3.51890	6.99990	19.52650	C48	-3.07530	5.92020	25.11120
C9	0.42250	9.47590	19.86450	H29	-1.53120	5.88270	19.04440	C49	-3.91010	5.27460	24.19600
H10	0.61540	9.51620	20.93900	H30	-2.72620	8.70500	18.56060	C50	-3.45730	5.02490	22.89640
C11	-1.31750	11.20530	20.49000	H31	-2.95760	7.28150	17.51160	C51	-2.18340	5.43760	22.48680
H12	-1.14070	10.94980	21.53120	H32	-4.36460	8.24390	18.02390	C52	-1.34460	6.05370	23.42130
C13	-2.28050	12.16170	20.15540	C33	-3.38110	7.86160	18.33400	C53	-1.78530	6.29760	24.72130
H14	-2.84060	12.66220	20.94240	C34	-4.15550	7.75570	20.62760	H54	-3.42020	6.11530	26.12480
C15	0.71300	7.53980	18.38750	H35	-3.56410	8.64420	20.84340	H55	-4.90690	4.95590	24.49480
O16	1.48950	8.66260	17.94760	H36	-5.17660	8.04360	20.33810	H56	-4.10390	4.49260	22.19910
C17	1.73460	9.50790	19.07320	H37	-4.18700	7.13430	21.52200	H57	-0.34790	6.34050	23.10590
C18	-0.10160	7.12590	17.16180	C38	-4.31140	5.79780	19.19250	H58	-1.12330	6.78600	25.43450
H19	0.59320	6.96690	16.33070	H39	-5.29640	6.08460	18.79760				
H20	-0.80010	7.91860	16.88520	H40	-3.78390	5.20540	18.44070				

(2*R*,3*S*)-TSelim

E(B3LYP-D3/6-31G*(dichloromethane)) = -1229.62227706789
E(B3LYP-D3/6-311+G**(dichloromethane)) = -1229.95705539704
G_{tot}(B3LYP-D3/6-31G*(dichloromethane)) = -1229.179452

Number of imaginary frequencies: 1 (-288.05)

C1	2.97470	7.59180	13.84280	N21	-0.59850	8.74780	16.96670	H41	0.43810	14.46280	19.35030
H2	2.73000	7.06290	12.92440	C22	0.63390	9.05540	19.68960	H42	2.53570	13.23550	18.80600
O3	-0.23240	6.31400	18.82030	C23	-1.91000	8.80220	17.64680	H43	2.61290	10.75110	19.03770
N4	1.84510	6.24400	17.86690	H24	-1.91330	9.63190	18.35350	H44	-1.46540	10.73280	20.35630
C5	3.33260	8.94340	13.79890	H25	-2.71260	8.95350	16.91140	H45	-1.55650	13.20360	20.14620
C6	2.92910	6.91680	15.06260	H26	-2.06170	7.86410	18.18200	O46	1.84830	8.51350	20.07540
H7	2.66050	5.86520	15.09270	C27	-0.60090	7.69080	15.93020	H47	-0.24870	8.65820	20.22490
C8	3.23530	7.58210	16.25870	H28	-0.88720	6.74080	16.38310	O48	3.21770	4.53840	17.23760
C9	3.16690	6.85980	17.59590	H29	-1.31930	7.94290	15.13710	H49	4.44910	5.44670	18.66990
H10	3.40210	7.55250	18.40750	H30	0.39750	7.60460	15.49720	H50	4.91280	5.65830	16.96040
C11	3.61440	8.92830	16.20340	C31	-0.25670	10.05120	16.35450	C51	1.89800	4.15580	19.24950
H12	3.87370	9.45120	17.12230	H32	-1.01290	10.32990	15.60780	H52	2.07390	3.07660	19.18660
C13	3.66030	9.60840	14.98150	H33	-0.20980	10.81720	17.12910	H53	2.66810	4.60480	19.88470
H14	3.94990	10.65650	14.95600	H34	0.71790	9.97530	15.86410	H54	0.92360	4.33940	19.70570
C15	1.93690	4.75180	17.83710	C35	0.47660	13.37940	19.44370	C55	0.88790	4.13110	16.91980
C16	4.06960	5.60980	17.65230	C36	1.65420	12.68830	19.13530	H56	1.08170	3.05810	16.81940
C17	0.77260	6.89850	18.39020	C37	1.69950	11.29960	19.25270	H57	-0.10850	4.27650	17.34210
H18	3.36090	9.47130	12.84820	C38	0.57050	10.57640	19.66070	H58	0.92830	4.59190	15.92870
C19	0.80820	8.40830	18.34610	C39	-0.59280	11.27780	19.99780				
H20	1.56880	8.88060	17.74290	C40	-0.64380	12.67150	19.88550				

(2*R*,3*R*)-TSelim

E(B3LYP-D3/6-31G*(dichloromethane)) = -1229.61635556270
E(B3LYP-D3/6-311+G**(dichloromethane)) = -1229.94983465887
G_{tot}(B3LYP-D3/6-31G*(dichloromethane)) = -1229.175265

Number of imaginary frequencies: 1 (-285.88)

C1	0.54310	7.99630	13.29600	N21	1.32210	6.27400	17.97670	C41	3.09200	8.94530	22.47680
H2	1.01870	7.51360	12.44480	C22	0.53590	8.69100	19.64930	H42	3.21330	7.72640	24.25830
O3	3.50260	8.31830	18.57080	H23	-0.47790	8.25610	19.75760	H43	1.21100	6.36760	23.66750
N4	2.62960	9.54180	16.85770	C24	2.10690	5.59760	19.02860	H44	-0.02140	6.82930	21.56490
C5	-0.82100	7.80160	13.54030	H25	1.60630	5.71820	19.98930	H45	2.70930	9.99330	20.62350
C6	1.30000	8.80550	14.14390	H26	2.20350	4.52560	18.80050	H46	3.95590	9.55310	22.73860
H7	2.35800	8.95810	13.95120	H27	3.09090	6.06240	19.08580	O47	0.55410	9.99230	19.17710
C8	0.70560	9.43060	15.25020	C28	1.95870	6.08880	16.65760	O48	3.58730	10.80740	15.21930
C9	1.52290	10.28440	16.20780	H29	2.99150	6.43930	16.70470	H49	2.42930	12.27370	16.16550
H10	0.87290	10.68790	16.98590	H30	1.95520	5.02620	16.37240	H50	1.87290	11.72190	14.56270
C11	-0.66470	9.24780	15.47530	H31	1.41690	6.66410	15.90320	C51	4.82190	8.86500	15.84660
H12	-1.13510	9.73390	16.32880	C32	-0.06740	5.77780	17.95060	H52	5.71060	9.27040	15.35120
C13	-1.42520	8.43440	14.62860	H33	-0.09200	4.69900	17.73960	H53	5.13340	8.23670	16.68330
H14	-2.48630	8.29430	14.82330	H34	-0.54040	5.96190	18.91800	H54	4.26290	8.25720	15.12900
C15	3.95560	10.01470	16.35210	H35	-0.62970	6.30180	17.17200	C55	4.68270	10.87680	17.39170
C16	2.31890	11.40180	15.50670	C36	2.67520	7.91960	23.33250	H56	5.60010	11.27630	16.94610
C17	2.51650	8.74140	17.95590	C37	1.55440	7.15500	22.99960	H57	4.05350	11.71230	17.71470
H18	-1.40790	7.16270	12.88390	C38	0.86570	7.41300	21.81150	H58	4.93600	10.27420	18.26560
C19	1.11430	8.30180	18.32360	C39	1.29450	8.41840	20.93640				
H20	0.38030	8.33550	17.53300	C40	2.40270	9.19440	21.28990				

(2*S*,3*R*)-TSelim

E(B3LYP-D3/6-31G*(dichloromethane)) = -1229.61246877480
E(B3LYP-D3/6-311+G**(dichloromethane)) = -1229.95141050488
G_{tot}(B3LYP-D3/6-31G*(dichloromethane)) = -1229.171833

Number of imaginary frequencies: 1 (-256.08)

C1	3.75270	9.02840	14.59300	H21	1.13070	7.87850	20.94820	C41	0.13900	10.93080	15.92860
H2	3.47910	8.55640	13.65220	H22	2.20510	9.27800	20.66800	C42	-0.23780	12.21530	15.54140
O3	0.51620	6.47570	18.74280	H23	0.87490	9.40180	21.84240	H43	-1.82900	13.67870	15.49840
N4	2.65970	7.20990	18.44080	C24	1.18150	8.96570	20.88380	H44	-3.46320	12.18300	16.62980
C5	4.32230	10.30490	14.59550	C25	-1.10840	8.97530	20.07530	H45	-2.78680	9.89590	17.31650
C6	3.51620	8.35710	15.79400	H26	-1.13160	7.88860	19.98860	H46	1.12900	10.54310	15.70140
H7	3.02130	7.39470	15.79020	H27	-1.41240	9.28760	21.08120	H47	0.47340	12.85850	15.02730
C8	3.85680	8.95500	17.00860	H28	-1.78220	9.41590	19.34130	O48	4.67120	6.12860	18.32540
C9	3.69180	8.25900	18.34780	C29	0.33250	10.92220	19.71940	H49	5.06880	7.48570	19.86690
H10	3.50710	9.02900	19.10470	H30	-0.03680	11.36260	20.65340	H50	5.85720	7.79970	18.29670
C11	4.41770	10.23960	17.00840	H31	1.36670	11.23700	19.55740	C51	2.82370	4.88150	17.49960
H12	4.67980	10.71450	17.95390	H32	-0.28260	11.25870	18.88510	H52	3.37130	3.93970	17.61080
C13	4.65380	10.91320	15.80940	H33	4.50100	10.82630	13.65760	H53	1.75440	4.68910	17.59830
H14	5.08980	11.91000	15.82370	C34	-0.27920	8.71440	17.04440	H54	3.02860	5.29300	16.50790
C15	3.28910	5.85650	18.57510	O35	0.53350	8.08440	16.14010	C55	3.09720	5.30240	19.99180
C16	4.93950	7.45130	18.77530	H36	-1.15260	8.12200	17.38670	H56	3.65190	4.36370	20.09430
C17	0.78680	8.77460	18.13000	C37	-1.53290	12.67760	15.80460	H57	3.46790	6.01140	20.73980
C18	1.30480	7.38490	18.44940	C38	-2.44800	11.83930	16.44320	H58	2.03660	5.12090	20.17750
N19	0.27650	9.43960	19.80480	C39	-2.06300	10.55210	16.83570				
H20	1.53610	9.52620	17.91080	C40	-0.76330	10.09310	16.60010				

(2*S*,3*S*)-Epoxide

E(B3LYP-D3/6-31G*(dichloromethane)) = -1055.18013639158
E(B3LYP-D3/6-311+G**(dichloromethane)) = -1055.46071366938
G_{tot}(B3LYP-D3/6-31G*(dichloromethane)) = -1054.853861

Number of imaginary frequencies: 1 (-14.99)

C1	4.86850	10.35850	16.59640	O16	3.41260	6.54140	18.67720	H31	4.98690	12.48910	16.24840
H2	5.81850	10.14410	16.11150	C17	2.84280	7.61300	19.42930	C32	-1.04070	9.25620	17.28390
O3	0.36520	6.52320	15.78370	C18	3.19440	5.57480	16.50850	O33	-1.32170	8.79200	15.94720
N4	1.70650	7.38670	17.42300	H19	3.84030	4.75090	16.83040	H34	-1.16900	10.33340	17.41520
C5	4.40320	11.67670	16.67530	H20	3.81360	6.35940	16.06410	C35	-2.15520	6.87150	20.68130
C6	4.11980	9.31490	17.14060	H21	2.48950	5.20820	15.75980	C36	-1.80410	8.21530	20.83930
H7	4.49230	8.29540	17.09450	C22	1.49390	5.07370	18.32690	C37	-1.47520	8.98760	19.72330
C8	2.89300	9.57310	17.76970	H23	0.99760	5.46730	19.21920	C38	-1.47980	8.41980	18.44060
C9	2.07510	8.44980	18.38360	H24	2.06860	4.18330	18.60240	C39	-1.84370	7.07530	18.28520
H10	1.17270	8.86520	18.83790	H25	0.72780	4.79740	17.59900	C40	-2.18030	6.30780	19.40180
C11	2.43810	10.89400	17.85140	C26	0.04440	8.82760	16.36410	H41	-2.41480	6.26960	21.54940
H12	1.49090	11.10770	18.34350	C27	0.71530	7.46650	16.49940	H42	-1.79130	8.66430	21.82990
C13	3.18750	11.94250	17.30820	H28	0.65570	9.59850	15.89040	H43	-1.21140	10.03690	19.84900
H14	2.81870	12.96360	17.37910	H29	2.15780	7.23640	20.20160	H44	-1.85680	6.64020	17.29150
C15	2.44010	6.11540	17.71860	H30	3.65520	8.16310	19.90850	H45	-2.46330	5.26580	19.27120

(2*R*,3*S*)-Epoxide

E(B3LYP-D3/6-31G*(dichloromethane)) = -1055.17957206028
E(B3LYP-D3/6-311+G**(dichloromethane)) = -1055.46265723653
G_{tot}(B3LYP-D3/6-31G*(dichloromethane)) = -1054.857347

Number of imaginary frequencies: 0

C1	-6.40660	8.38600	22.51350	O16	-4.51390	3.53310	20.55240	H31	-7.88840	9.91070	22.13190
H2	-6.25580	8.67480	23.55150	C17	-5.64820	4.39740	20.56900	C32	-0.27680	6.59510	19.77800
O3	-3.19770	7.57390	19.37410	C18	-3.28540	4.22730	22.53200	O33	-0.65920	7.80110	20.45790
N4	-3.70210	5.66600	20.50950	H19	-3.09580	3.19550	22.84600	H34	-0.49980	6.60580	18.70930
C5	-7.32220	9.08000	21.71670	H20	-4.21590	4.56480	22.99870	C35	3.30910	4.54700	20.94690
C6	-5.68030	7.32250	21.97840	H21	-2.47130	4.85630	22.90570	C36	2.69660	4.25750	19.72480
H7	-4.96050	6.79560	22.59920	C22	-2.16750	3.65220	20.32130	C37	1.54350	4.94490	19.34000
C8	-5.85290	6.94100	20.64150	H23	-2.22350	3.79790	19.23870	C38	0.98970	5.92010	20.18020
C9	-5.10670	5.75050	20.06890	H24	-2.18130	2.57820	20.53290	C39	1.60940	6.21080	21.40300
H10	-5.13820	5.79050	18.97360	H25	-1.22290	4.05480	20.69260	C40	2.76500	5.52770	21.78270
C11	-6.76640	7.64210	19.84930	C26	-1.44670	6.63060	20.69330	H41	4.20960	4.01450	21.24480
H12	-6.89940	7.36060	18.80650	C27	-2.84790	6.66040	20.12290	H42	3.11880	3.50000	19.06850
C13	-7.50040	8.70450	20.38330	H28	-1.31990	6.20760	21.68840	H43	1.07060	4.72350	18.38480
H14	-8.20580	9.24340	19.75450	H29	-6.41300	3.97390	19.91390	H44	1.18810	6.98140	22.04390
C15	-3.38100	4.28290	21.00170	H30	-6.06110	4.49430	21.58270	H45	3.24440	5.76240	22.73040

(2*R*,3*R*)-Epoxide

E(B3LYP-D3/6-31G*(dichloromethane)) = -1055.17847898574

E(B3LYP-D3/6-311+G**(dichloromethane)) = -1055.46022603155

G_{tot}(B3LYP-D3/6-31G*(dichloromethane)) = -1054.854915

Number of imaginary frequencies: 0

C1	0.14270	8.37750	13.78380	O16	3.88610	11.19540	16.81820	H31	0.70540	6.75440	17.32800
H2	0.43690	7.66240	13.01830	C17	2.67610	11.29980	16.07730	C32	0.58870	7.64070	19.35590
O3	3.35530	7.14540	17.78380	C18	4.87830	9.15320	15.91680	H33	-0.10050	6.85180	19.66560
N4	2.72580	9.26530	17.19370	H19	5.87720	9.59600	15.83820	C34	2.93190	9.35170	22.52720
C5	-1.16930	8.85930	13.82950	H20	4.35620	9.30400	14.96670	C35	2.62670	7.98700	22.52610
C6	1.08090	8.81060	14.72210	H21	4.97260	8.08020	16.09920	C36	1.85610	7.44140	21.49840
H7	2.09750	8.42560	14.68420	C22	4.88340	9.72250	18.39290	C37	1.39150	8.25410	20.45590
C8	0.71800	9.72230	15.72270	H23	4.29300	10.17250	19.19480	C38	1.69080	9.62170	20.46710
C9	1.73510	10.25230	16.71420	H24	5.82680	10.26870	18.28930	C39	2.45720	10.16820	21.49770
H10	1.21440	10.69990	17.56530	H25	5.09620	8.68260	18.64500	H40	3.52980	9.77670	23.33020
C11	-0.59830	10.19590	15.76530	C26	2.47740	8.00220	17.62080	H41	2.98600	7.34820	23.32970
H12	-0.89280	10.89140	16.54890	H27	2.29620	12.32030	16.17050	H42	1.61550	6.37960	21.50260
C13	-1.53670	9.77180	14.82190	H28	2.83560	11.07550	15.01340	H43	1.31060	10.25530	19.67360
H14	-2.55730	10.14720	14.86820	H29	-1.90170	8.52180	13.09930	H44	2.67940	11.23340	21.49750
C15	4.11970	9.80780	17.07660	C30	1.05850	7.57960	17.94860	O45	0.05540	8.52160	18.34560

(2*S*,3*R*)-Epoxide

E(B3LYP-D3/6-31G*(dichloromethane)) = -1055.18291837702

E(B3LYP-D3/6-311+G**(dichloromethane)) = -1055.46507631373

G_{tot}(B3LYP-D3/6-31G*(dichloromethane)) = -1054.860355

Number of imaginary frequencies: 0

C1	4.84290	8.78590	14.70580	O16	3.94790	6.53920	19.76650	H31	5.54900	10.63540	13.84140
H2	5.04140	8.19640	13.81290	C17	4.60090	7.57930	19.05300	C32	-0.47160	8.71450	16.64080
O3	0.77630	6.06560	17.07410	C18	3.56020	4.84760	18.04260	O33	0.23240	8.31040	15.45560
N4	2.55340	7.12810	18.05050	H19	3.88170	4.01950	18.68310	H34	-1.14320	7.95040	17.03490
C5	5.12710	10.15510	14.72130	H20	4.42530	5.20970	17.47800	C35	-1.71130	12.80690	17.03970
C6	4.29860	8.16920	15.83310	H21	2.80740	4.48690	17.33700	C36	-2.29690	11.81250	17.82800
H7	4.07070	7.10630	15.80930	C22	1.83650	5.46950	19.80750	C37	-1.91190	10.47830	17.67340
C8	4.02420	8.91660	16.98640	H23	1.40660	6.31280	20.35700	C38	-0.93140	10.13060	16.73550
C9	3.47810	8.26780	18.24580	H24	2.25450	4.75650	20.52570	C39	-0.35600	11.12980	15.93790
H10	2.99620	9.02950	18.87200	H25	1.05670	4.97870	19.22500	C40	-0.74350	12.46100	16.09060
C11	4.31010	10.28770	16.99630	C26	0.98300	8.40560	16.67220	H41	-2.00910	13.84610	17.16170
H12	4.09540	10.87750	17.88680	C27	1.43720	7.09160	17.26880	H42	-3.05420	12.07540	18.56320
C13	4.86260	10.90420	15.87060	H28	1.65810	9.25430	16.71000	H43	-2.36900	9.70510	18.28880
H14	5.07900	11.97020	15.89000	H29	5.07840	8.25160	19.76980	H44	0.38810	10.85550	15.19430
C15	2.96220	5.96330	18.90400	H30	5.36590	7.18180	18.37160	H45	-0.29100	13.23030	15.46840

Trimethylamine

E(B3LYP-D3/6-31G*(dichloromethane)) = -174.47794816150

E(B3LYP-D3/6-311+G**(dichloromethane)) = -174.53173369370

G_{tot}(B3LYP-D3/6-31G*(dichloromethane)) = -174.383657

Number of imaginary frequencies: 0

```
N1   -0.67810    8.72130  16.78190
C2   -1.93900    8.90300  17.49820
H3   -1.82810    9.68540  18.25850
H4   -2.77750    9.19200  16.83230
H5   -2.21730    7.97200  18.00650
C6   -0.78940    7.65230  15.79160
H7   -1.07780    6.71590  16.28410
H8   -1.54040    7.86780  15.00450
H9    0.17820    7.49410  15.30050
C10  -0.24840    9.96950  16.15610
H11  -0.96850   10.34600  15.40130
H12  -0.12340   10.74640  16.91980
H13   0.71720    9.82410  15.65710
```
